# Bumblebee responses to variation in pollinator‐attracting traits of *Vicia faba* flowers

**DOI:** 10.1002/ece3.10617

**Published:** 2023-11-10

**Authors:** Emily J. Bailes, Jake Moscrop, Sarah Mitchell, Matthew Dorling, Tom Wood, Jane Thomas, Beverley J. Glover

**Affiliations:** ^1^ Department of Plant Sciences University of Cambridge Cambridge UK; ^2^ NIAB Cambridge UK

**Keywords:** attractive traits, *Bombus terrestris*, corolla‐tube length, flower size, *Vicia faba*, volatile organic compounds

## Abstract

Adaptations that attract pollinators to flowers are central to the reproductive success of insect‐pollinated plants, including crops. Understanding the influence of these non‐rewarding traits on pollinator preference is important for our future food security by maintaining sufficient crop pollination. We have identified substantial variation in flower shape, petal size, corolla‐tube length, petal spot size and floral volatile compounds among a panel of 30 genetically distinct lines of *Vicia faba*. Using this variation, we found that *Bombus terrestris* was able to distinguish between natural variation in petal spot size, floral volatile emissions and corolla‐tube length. Foragers showed some innate preference for spotted flowers over non‐spotted flowers and preferred shorter corolla‐tube lengths over longer tubes. Our results suggest that some floral traits may have significant potential to enhance pollinator attraction to *V. faba* crops, particularly if paired with optimised rewards.

## Graphical abstract

Bumblebees show preferences for, and ability to distinguish between, extremes of *Vicia faba* floral trait variation that is not directly rewarding, with implications for pollination services and crop breeding.

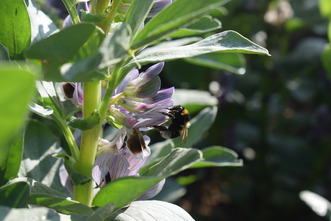

## 1 INTRODUCTION

Adaptations to attract pollinators are thought to have played an important role in the evolution and diversification of the angiosperms (flowering plants; Christenhusz & Byng, 2016; Dodd et al., 1999; Stebbins, 1981). Whilst pollinators usually visit flowers to access a reward, many traits increase the attractiveness of flowers without necessarily having any direct relationship to the reward available—they act as attractive signals to potential pollinators. At the flower level, traits such as petal size, shape, colour, patterning and floral scent can be viewed as traits of attraction rather than directly rewarding traits. Although the reward itself will attract pollinators, here we use the term ‘attractive traits’ to refer to non‐rewarding traits. In some cases, variations in attractive traits within a species are correlated with the reward produced by flowers (honest signalling); for example flower size is often found to be a reliable indication of the quantity of nectar produced by a flower (e.g. Conner & Rush, 1996; Eisen et al., 2023; Galen & Newport, 1987). Other traits, such as within‐species variation in colour or scent, can be indicators of floral reward, but in other cases they are not correlated (Eisen et al., 2023; Essenberg, 2021; Haber et al., 2019; Knauer & Schiestl, 2015).

Both visual and olfactory stimuli are important in pollinator attraction, and in some cases, they have been shown to work synergistically (Klahre et al., 2011). For example, both olfactory and visual cues are needed to stimulate the hawkmoth *Manduca sexta* to feed on *Datura wrightii* (Raguso & Willis, 2005). Floral cues can vary in how they attract pollinators to the flower at different distances. For example, floral volatiles can act as attractants and aid in flower location from long distances, but they have also been shown to trigger feeding behaviours at close range (Cunningham et al., 2004; Raguso & Willis, 2005). Flower colour acts at a shorter range than scent, as a general attractant, providing contrast against green vegetation, but detailed floral patterning can improve insect foraging efficiency at an even closer range, only being perceptible to an insect very close to the flower (Leonard & Papaj, 2011). In some cases, these traits can attract certain pollinating species but not others, often due to differences in pollinator sensory systems or morphology. The bright red flowers of *Mimulus cardinalis* are clearly visible to its hummingbird pollinators but less salient to the eyes of bees, which contain different photoreceptors (Chittka & Waser, 1997; Forrest & Thomson, 2009; Herrera et al., 2008). Similarly, the scent of night‐flowering *Petunia axillaris* is specifically attractive to its pollinating moth, particularly in combination with white‐coloured flowers (Klahre et al., 2011).

Understanding how floral traits influence pollinator behaviour is a key strategy to make global food security more sustainable (Bailes et al., 2015; Carruthers et al., 2017; Palmer et al., 2009; Prasifka et al., 2018). Many important crops are animal‐pollinated, and pollinator visitation is required to differing extents to achieve maximum yield (depending on their breeding system and degree of self‐compatibility; Klein et al., 2007). In addition to overall yield, visitation by pollinators can improve the yield stability, quality and overall value of animal‐pollinated crops (Bishop et al., 2022; Garratt et al., 2014; Gazzea et al., 2023). In general, increased visitation rates to crops by honey bees are associated with higher seed set, up until an optimum level (Rollin & Garibaldi, 2019). Given that heritable variation in floral traits of crop species is present (e.g. Dowell et al., 2019; Hughes et al., 2020; Sundramoorthy et al., 2020) and that flower traits are important for plant–pollinator interactions, selecting from this variation has great potential to increase pollinator attraction to crops and benefit global food production.

In agricultural species, research indicates that a wide range of attractive flower traits are associated with higher pollinator visitation rates, although it can be difficult to disentangle these from differences in floral reward. In *Fragaria* × *ananass* (strawberry) and *Vaccinium corymbisum* (blueberry), a greater quantity of floral volatiles has been associated with increases in pollinator visitation, but floral reward was not quantified (Klatt et al., 2013; Rodriguez‐Saona et al., 2011). In *V. corymbisum*, flowers with wider corollas (representing greater accessibility of the reward) have also been correlated with higher honeybee visits (Courcelles et al., 2013). Flower colour has been linked to differences in visitation rates to *Medicago sativa* (alfalfa), with pollinator species‐specific colour preferences (Bauer et al., 2017). Where floral reward has been quantified, attractive floral traits still contribute to overall visitation rates. In *Solanum lycopersicum* (tomato), specific elements of the floral volatiles were associated with increased bee visitation but not an increased floral reward (Morse et al., 2012). A shorter corolla‐tube length has been linked to higher visitation rates and outcrossing (a proxy for visitation rates) in *Helianthus annuus* (sunflower) and *Vicia faba* (field bean), respectively (Mallinger & Prasifka, 2017; Suso et al., 2005). In *H. annuus*, nectar production also contributed to higher visitation rates, and in *V. faba* corolla‐tube length was only important when flowers produced nectar (Mallinger & Prasifka, 2017; Suso et al., 2005). Changes in the standard petal dimensions have also been associated with increased outcrossing in *V. faba* (Suso et al., 2005; Suso & Maalouf, 2010). However, further studies investigating natural variation in attractive traits and their influence on pollinator behaviour are crucial for success in breeding for higher‐yielding animal‐pollinated crops.

**FIGURE 1:**
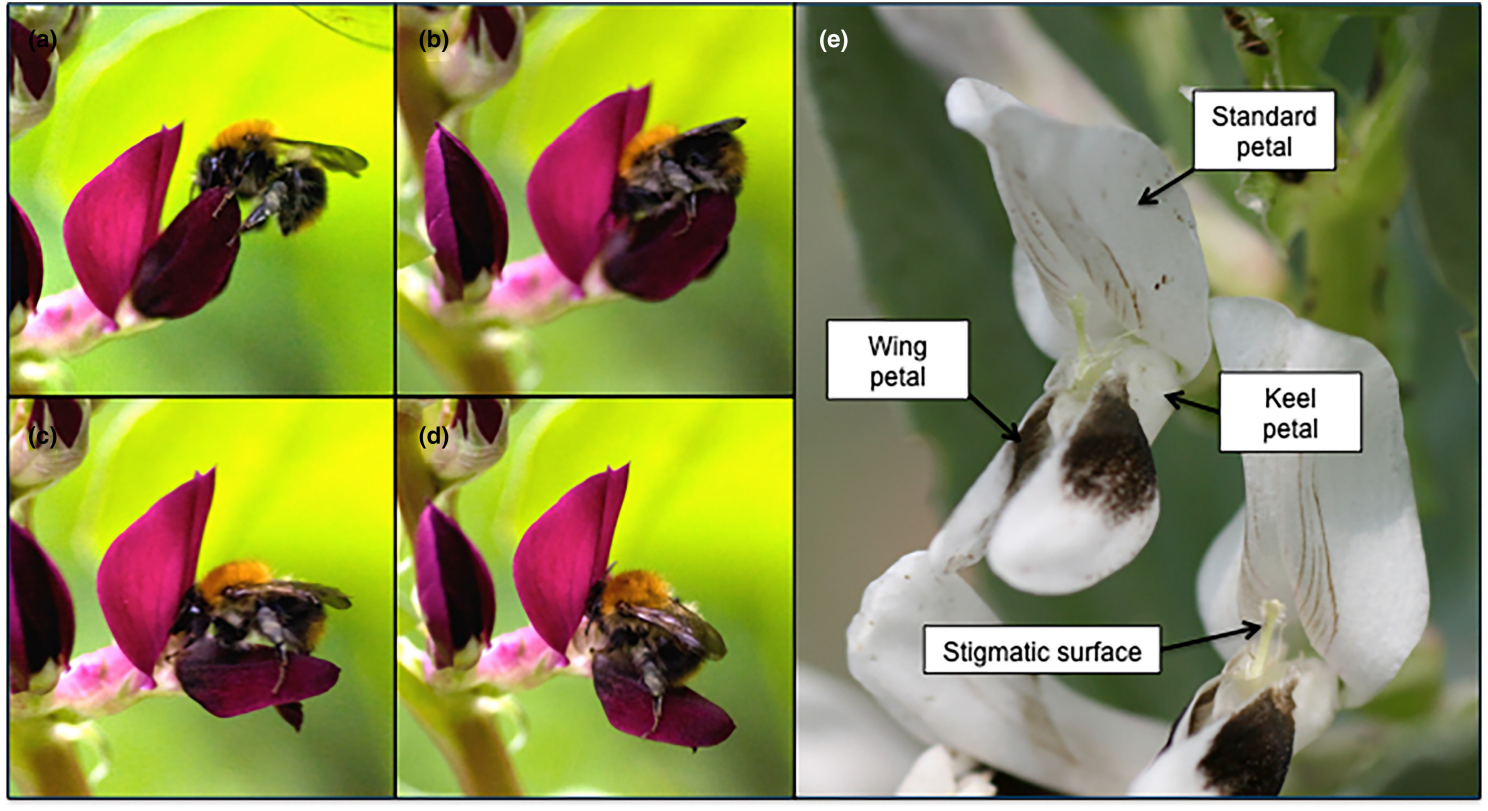
*Vicia faba* flowers being pollinated (‘tripped’). *Bombus pascuorum* is pictured landing on and tripping a flower of *V. faba* NV706 by pushing the keel‐wing complex downwards (a–d). When *V. faba* flowers have been tripped, they will temporarily reveal the brush‐like stigmatic surface (e). Photos taken by E.J.B in the UK.

*Vicia faba* (Figure 1) is an ideal system in which to measure intraspecific floral trait variation and its effect on floral attractiveness. The crop benefits from bee pollination, with an average of ~33% of yield lost in the absence of bees and an increase in yield stability of ~19% when pollinators are present (Bishop et al., 2022; Bishop & Nakagawa, 2021). Depending on the locality grown, the pollinating species vary, with *Bombus* spp., *Apis mellifera* and *Eucera* spp. commonly reported (Aouar‐Sadli et al., 2008; Hutchinson et al., 2021; Lundin, 2023; Marzinzig et al., 2018; Pierre et al., 1999). *Vicia faba* is usually grown as a single variety, flowering only for a limited period. Since the absence of bees reduces yield, it follows that identification of *V. faba* lines that are most attractive to foragers visiting co‐flowering wild plants or other crops could enhance total crop yield by maximising pollination within the flowering window, although this will vary between cultivars (Bishop et al., 2020). A previous study found substantial variation in the amount of nectar and pollen produced by flowers between a large panel of inbred *V. faba* lines (Bailes et al., 2018). However, variation in attractive floral traits has only been explored in a small number of distinct genotypes (e.g. Suso et al., 2008), in populations of mixed genotypes (e.g. Suso & del Río, 2014) or for individual traits such as floral volatiles (e.g. Griffiths et al., 1999).

Here we investigate multiple attractive traits of distinct *V. faba* lines and the impact of a subset on bumblebee preference. We determined whether there are differences in the colour and shape of flowers across a panel of 30 inbred lines of *V. faba* in which floral reward has previously been quantified. We examined the volatile organic compounds produced by the flowers of two lines. We use bee behavioural experiments to determine if *Bombus terrestris* can detect variation in some of these floral traits—scent, petal spot presence and size and corolla‐tube length—and whether they exhibit a preference when the traits are not associated with differences in floral reward.

## 2 MATERIALS AND METHODS

### 2.1 Study species and growth conditions

Thirty lines of *V. faba*, which had been self‐pollinated for a minimum of five generations (with the exception of NV706) to minimise genetic variation within a line, were used for this study. Replicate plants of each line were grown in insect‐proof, temperature‐controlled glasshouses between September and May of 2013 and 2015. Plants within a line were grown across multiple months/years to control for any environmental variation. Plants for headspace collections were grown simultaneously. Plants were grown in 1 L pots of Levington® M3 Pot and Bedding Compost. Glasshouse conditions were maintained at 18–25°C with 16–18 h of daylight. When daylight levels fell below 20,000 lux, 10,000 lux high‐pressure sodium lights were activated. The predatory mite *Amblyseius andersoni* was used to control thrips (Thysanoptera) levels.

### 2.2 Measurement of floral traits

Fresh open flowers (Stage 4–5 according to Osborne et al., 1997) were used for all floral trait measurements. *Vicia faba* flowers comprise three petal types (Figure 1): the large standard petal at the top of the flower and two lateral wing petals, which surround the keel pocket (formed of two fused ventral petals). When a bee legitimately visits a flower, it lands on the wing petals and pushes into the flower to access the pollen and nectar. Therefore, measurements have focused on the standard and wing petals, as these are the petals the pollinator interacts with most visually and physically.

Flower morphology and colour were quantified for all 30 lines. From this, two lines with a notable difference in colour patterning (spotted NV641 and non‐spotted NV676) but similar morphology were investigated for their floral volatile organic compounds before carrying out bee behaviour experiments.

#### 2.2.1 Colour

Hymenoptera such as bees are able to perceive a different range of wavelengths than humans (Menzel & Blakers, 1976; Peitsch et al., 1992). Therefore, to determine bee‐perceptible differences in colour, we measured the reflectance spectra of flowers and used these to infer how bees would perceive flower colour. Reflectance spectra were measured using a spectrophotometer (Ocean Optics 2+) with a 10 ms integration time and black background corrected for. Samples were illuminated with a Deuterium‐Halogen light source (Ocean Optics DH 2000) and analysed with SpectraSuite software (version 1.0, Ocean Optics). Measurements were taken of the standard petal adaxial surface, the tip of the wing petal abaxial surface and the centre of the wing petal abaxial surface (where the spot was located, if present). The average reflectance spectra of 10–20 flowers per line (each from a different plant; see Tables S9–S11 for exact *n* values) were used to calculate the excitation of bee UV, blue and green photoreceptors relative to a green background following Chittka (1992).

#### 2.2.2 Morphological measurements from imaged flowers

Flowers were imaged to quantify morphology and spot size (Figure S1). The five measurements chosen were selected based on preliminary analyses of 14 measurements from 10 lines that suggested lower levels of environmental variation and measurement error and low correlation between traits whilst describing key aspects of the flower for pollinator interaction—flower size and the shape of wing petals (which bees land on).

Side‐on images of intact flowers were used to calculate standard petal height and corolla‐tube length. Images of flattened wing petals (abaxial side up) were used to calculate the wing area and the size of the wing spot relative to the overall size of the wing petal. The ratio between wing length and width was calculated from an unflattened petal. Dimensions were calculated in Fiji (ImageJ v2.0.0—http://fiji.sc/Fiji; see Data S1 for details). Three flowers (one per week) were measured per plant from a minimum of five plants per line (exact numbers—Table S2 and Figure S2).

#### 2.2.3 Volatile analysis

Dynamic headspace trapping was used to collect volatile organic compounds (VOCs) from the flowers of NV641 and NV676 in a method adapted from Beale et al. (2006; Figure S3). Each sample was collected from eight open flowers (stages 4–5) from 2 to 3 plants over a 24‐h period. Plants were not reused between collections. A total of six and four successful collections were made from NV641 and NV676, respectively. GC–MS was used to identify VOCs in each sample (see Data S1). Each compound (not present in flower‐free negative controls) was tentatively identified using the National Institute of Standards and Technology (2005) mass spectra database. The identity of ocimene was verified by collecting VOCs from flowers plus 2 μL of ocimene (Sigma Aldrich, mixture of isomers ≥90% purity). Linalool was additionally verified using an authentic standard.

### 2.3 Bee behavioural experiments

Initial experiments focused on lines NV641 and NV676, which were similar in morphology but differed in the absence/presence of spots and their floral VOCs. We were interested in spot presence as this trait is associated with the nutritional content of the crop (Hou et al., 2018), and we wanted to determine if this would lead to any trade‐offs with bee preference. Additional experiments explored more subtle variation in spot size, choosing the extremes of our dataset as bees were most likely to be able to discriminate between them.

Additionally, from the morphological variation, we chose corolla‐tube length to investigate further, as it will affect the accessibility of the nectar reward and has been reported to be important in the field for bee visitation to sunflowers (Mallinger & Prasifka, 2017; Portlas et al., 2018).

Experiments were carried out in a 0.3 × 0.75 × 1.12 m plywood flight arena with a clear UV‐transparent Plexiglass lid using *B. terrestris audax* (Agralan). *Bombus terrestris* was used in experiments as this is the only commercially available bumblebee species and is a commonly observed flower visitor that is known to improve *V. faba* yield (e.g. Bishop et al., 2016; Hutchinson et al., 2021; Marzinzig et al., 2018). All ‘flowers’ used in these experiments, including the training phases, were artificial, with the exception of the flowers of NV641 and NV676 used in experiments 1, 2 and 3. ‘Flower’ types are summarised in Table 1. Behavioural experiments consisted of a training phase, where all bees were allowed to visit artificial training flowers loaded with *~*30% w/w sucrose solution. This was followed by the trial phase, where a single forager was allowed into the foraging arena. The experimental setup for the six experiments is summarised in Table 1. Experiments contained equal numbers (4 or 5; Table 1) of each flower type; for example four spotted flowers and four non‐spotted flowers. After a bee had fed from a flower, the reward was replenished, and the flower was repositioned to eliminate positional effects. Artificial flowers and towers were cleaned with 30% v/v ethanol between foraging bouts, with the exception of spot‐size models, which were cleaned with distilled water to avoid removing the spot. Flowers for experiments 1–3 came from multiple plants (3–6) per experimental run (bee). Plants were reused between experiments, but the same combination of plants were not used in every run. For experiments 4–6, the same set of artificial flowers were reused between experimental runs.

**TABLE 1:** Summary of the experimental setup for the six bee behavioural experiments carried out in this study. Images of artificial flowers can be seen in Figure 6.

Experiment	1	2	3	4	5	6
Question	Can bees perceive differences in the scent of flowers from NV641 and NV676?	Do bees prefer the scent of flowers from NV641 or NV676?	Do bees prefer flowers with wing petal spots?	Can bees perceive the difference between large and small spotted petal models?	Do bees prefer large or small spotted wing petals?	Do bees prefer flowers with shorter corolla tubes?
Flower type and number used	Black tower containing 3 flowers of NV641 or NV676 (4 of each type)	Black tower containing 3 flowers of NV641 or NV676 (4 of each type)	7‐cm clear pot with a flower of NV641 (spotted) or NV676 (non‐spotted) on top (4 of each type)	Large or small spotted epoxy *V. faba* flower model (4 of each type)	Large or small spotted epoxy *V. faba* flower model (4 of each type)	Blue or purple disk with the base of a cut‐down pipette tip to 12 mm or 16 mm long at its centre (5 of each type)
Reward	*Reward*: 10 μL of 40% w/w sugar solution *Distractor*: 0.12% w/v quinine solution	7 μL of 40% w/w sugar solution	10 μL of 30% w/w sugar solution	*Reward*: 10 μL of 40% w/w sugar solution *Distractor*: 0.12% w/v quinine solution	10 μL of 40% w/w sugar solution	10 μL of 30% w/w sugar solution
Training flowers	Empty towers	Empty towers	Grey disks on top of on 7‐cm clear pot	0.2 mL PCR tube on top of a 7‐cm clear pot attached using a dowel	0.2 mL PCR tube on top of a 7‐cm clear pot attached using a dowel	As above, with a grey corolla and a 14‐mm centre
Choices recorded	80 landings (contact with flower)	10 landings (contact with flower)	10 landings (contact with flower)	200 landings (contact with flower)	10 landings (contact with flower)	100 landings and/or successful feedings
*n* bees	10^a^	16	10	20^a^	50	10/15^b^, respectively

^a^ Half of the bees were tested with flower type 1 rewarded, and half with flower type 2 rewarded.

^b^ The flowers fed from and landed on were recorded for five bees with short‐tubed purple flowers and five bees with short‐tubed blue flowers; the flowers fed from for an additional five bees with short‐tubed blue flowers were recorded.

#### 2.3.1 Experiment 1: Can bees perceive differences in the scent of flowers from NV641 and NV676?

Lines NV641 and NV676 were found to have different scents. Before assessing whether bees prefer the scent of one line over another, we tested whether they were capable of perceiving differences in the scent of flowers from lines NV641 and NV676. In each replicate, the scent of one line (e.g. NV641) was paired with 10 μL of 40% w/w sucrose reward and the other line (e.g. NV676) with 10 μL of 0.12% w/v quinine distractor solution. Foragers are unable to differentiate between sucrose solution and quinine solution except by the bitter taste. However, if quinine is paired with a cue that they can perceive before feeding, they will learn to avoid quinine‐containing flowers (Groen et al., 2016; Whitney et al., 2008; Extended data figure 9 within Moyroud et al., 2017). We used an experimental design that removed all visual signals, as described by Groen et al. (2016). Three flowers of one line were placed under each of four black towers with a mesh top, which allowed volatiles to escape. The mesh was topped with a cup into which a sucrose reward or quinine punishment was placed (Figure S4). A total of 80 choices (landing on the gauze on the top of the tower) of each of 10 scent‐naïve bees were recorded. Equal numbers of bees were tested with NV676 and NV641 as the rewarded scents.

#### 2.3.2 Experiment 2: Do bees prefer the scent of flowers from NV641 or NV676?

Having established that bumblebees could distinguish between the scents of the two lines, we explored whether they preferred one over the other. Sixteen foragers were given the choice between the same eight towers as in experiment 1, but with all towers equally rewarded with 7 μL of 40% w/w sugar. As bees can quickly learn to associate a scent with a reward, only the first 10 landings made by a forager were recorded as their naïve preference.

#### 2.3.3 Experiment 3: Do bees prefer flowers with or without wing petal spots?

To examine if bees have a preference for wing petal spots, lines NV641 (spotted) and NV676 (non‐spotted) were chosen due to their similar size and the absence of a naïve preference for their scent by bees (established in experiment 2). Ten foragers were presented with four fresh single flowers of each type on top of 7‐cm‐high clear pots. Nectar was removed from the flowers using microcapillaries. A pipette tip was then inserted into the flower and loaded with 10 μL of a 30% w/w sugar solution, so that both flower types were equally rewarded. As for experiment 2, the first 10 choices (contact with the flower) made by each forager were recorded.

#### 2.3.4 Experiment 4: Can bees perceive differences in wing spot petal size?

To test whether bees could perceive differences in spot size, we used model flowers that represented extremes of spot size variation present in our dataset of real flowers. Real flowers were not used because these flowers were not similar in size. Twenty foragers were presented with four small (20% petal area) and four large (60% petal area) spotted flower models. In a similar setup to experiment 1, one spot size was paired with a 40% w/w sugar solution reward and the other with quinine hemisulphate solution (0.12% w/v) as a distractor. One hundred visits were recorded for each forager. Equal numbers of foragers were tested with the small or large spot size model rewarded.

#### 2.3.5 Experiment 5: Do bees show preference between large or small wing petal spots?

Having established that bumblebees could distinguish between large and small petal spots in experiment 4, we explored if they had an innate preference for one over the other using the same models. Fifty spot‐naïve foragers were individually presented with four large and four small spotted model flowers loaded with a 40% w/w sugar solution. The first 10 choices (contact with a flower) made by each forager were recorded.

#### 2.3.6 Experiment 6: Do bees prefer flowers with shorter corolla tubes?

To test whether bees could perceive differences in corolla‐tube length, we used artificial flowers that represented extremes present in our dataset of real flowers. As bees could not easily determine corolla‐tube length from a distance, different length tubes were paired with a colour cue, and we tested if the visitation pattern changed as bees learned to associate the two flower traits. Artificial flowers were created by placing a pipette tip of either 12 or 16 mm length at the centre of a disc made of pigmented epoxy resin (blue or purple). Both flower types were rewarded with 10 μL of 30% w/w sucrose solution at the base of each ‘corolla tube’. As training, foragers were presented with grey discs with a 14‐mm‐long ‘corolla tube’. After training, 100 choices (100 landings and 100 feeding events) between the two flower types were recorded for each bee. Five bees were assigned short‐tubed blue discs, and five were assigned short‐tubed purple disks. Additionally, data for 100 choices (only feeding events) made by five bees assigned short‐tubed blue discs were recorded.

### 2.4 Statistical methods

All statistical analyses were carried out in R version 3.4.1 (R Core Team, 2017), the code for these analyses are given in Glover et al. (2023).

#### 2.4.1 Differences in floral traits

To determine whether lines had significantly different floral morphologies or spot sizes, linear mixed models with a Gaussian distribution were run using the package ‘nlme’ (Pinheiro et al., 2017). Models were fitted using maximum likelihood estimation to account for unequal sample sizes, with sum contrasts and without an intercept. Model selection was carried out using the MuMIn package (Bartoń, 2017), testing all nested models of the full model (Table 2) containing the fixed categorical factors of *Line* (the genetic line the flower was from), *Month* (the month in which a measurement was taken; specified as fixed to test environmental effects were having a significant impact on flower traits), *Replicate* (equivalent to the week of flowering that a flower was photographed; specified as fixed as there were only three levels). *Plant* (unique ID for independent plants) was included as a random effect in all models tested to account for multiple measures of the same plant. All explanatory variables were categorical. Wing shape was transformed to *Ln(WgShape)* to satisfy the assumption of homogeneity of variance with the predicted value. To avoid zero bias of the data sets, non‐spotted lines NV175, NV643, NV644 and NV676 were removed from analyses of *%SpotSize*, and the ‘closed flower’ mutant line NV658 was removed from analyses of *StndHeight*. The data reported in the results section are for the model with the lowest AICc (Akaike Information Criterion corrected for small sample sizes) value. The AICc values for all iterations of the models are given in Tables S3–S7. The best model‐predicted flower morphologies were highly similar to those averaged over models with a weight greater than 0.05 (Data S1). The significance of the fixed effects was tested using likelihood ratio tests of nested models. To determine if individual lines had significantly different morphologies, a Tukey–Kramer test was carried out on the best model for each response variable using the ‘emmeans’ package (Lenth, 2018). Variance components were estimated using the ‘VCA’ package (Schuetzenmeister & Dufey, 2017), using a model where all factors were specified as random.

**TABLE 2:** The full and final linear mixed models reported in this study.

Dependent variable	Full fixed effects	Fixed effects included in best model	Random factors	Lines included
StandardHeight	Line, month, replicate	Line, month	Plant	29^a^
WgArea		Line, month, replicate		30
Ln(WgShape)		Line, month		30
Corolla‐tube length		Line, month		30
%SpotArea		Line, month, replicate		26^b^

^a^ Line NV658 was excluded to remove zero bias (see section 2.4 Materials and Methods).

^b^ Lines NV175, NV643, NV644 and NV676 were excluded as they lacked a petal spot.

#### 2.4.2 Bee experiments

For experiments conducted over a large number of choices (experiments 1, 4 and 6 in Table 1), we used a logistic regression with a binomial distribution and logit link function in the package ‘lme4’ (Bates et al., 2015). This model is designed to be used with binomial data and has previously been used for bumblebee learning data (Foster et al., 2014; Moyroud et al., 2017). Correct choices (short tubed/rewarded scent/spot size visited) were coded as 1, and incorrect choices 0. All models included ‘bee_ID’ (the individual tested) as a random factor to account for pseudoreplication. The continuous fixed effect ‘choice’ (flower choice number) was used to test for learning—where the probability of a correct choice was significantly different depending on the choice number, learning has taken place. Corr_line (which flower type was coded correct) was included as a fixed categorical factor when significant in the full model. The significance of the fixed effects was tested using likelihood ratio tests of nested models.

To test for innate preferences for floral scent, presence/absence of a wing petal spot and spot size (Experiments 2, 3 and 5 in Table 1), two tests were used:

- A binomial test to analyse if the first choice made by each of the bees was significantly different from the expected probability of 0.5.
- A two‐tailed *t*‐test was used to determine if the first 10 choices made by foragers had a significantly different number of choices than those expected by chance (five choices for each flower type).

## 3 RESULTS

### 3.1 The colour of *V. faba* flowers in bee visual space

**FIGURE 2:**
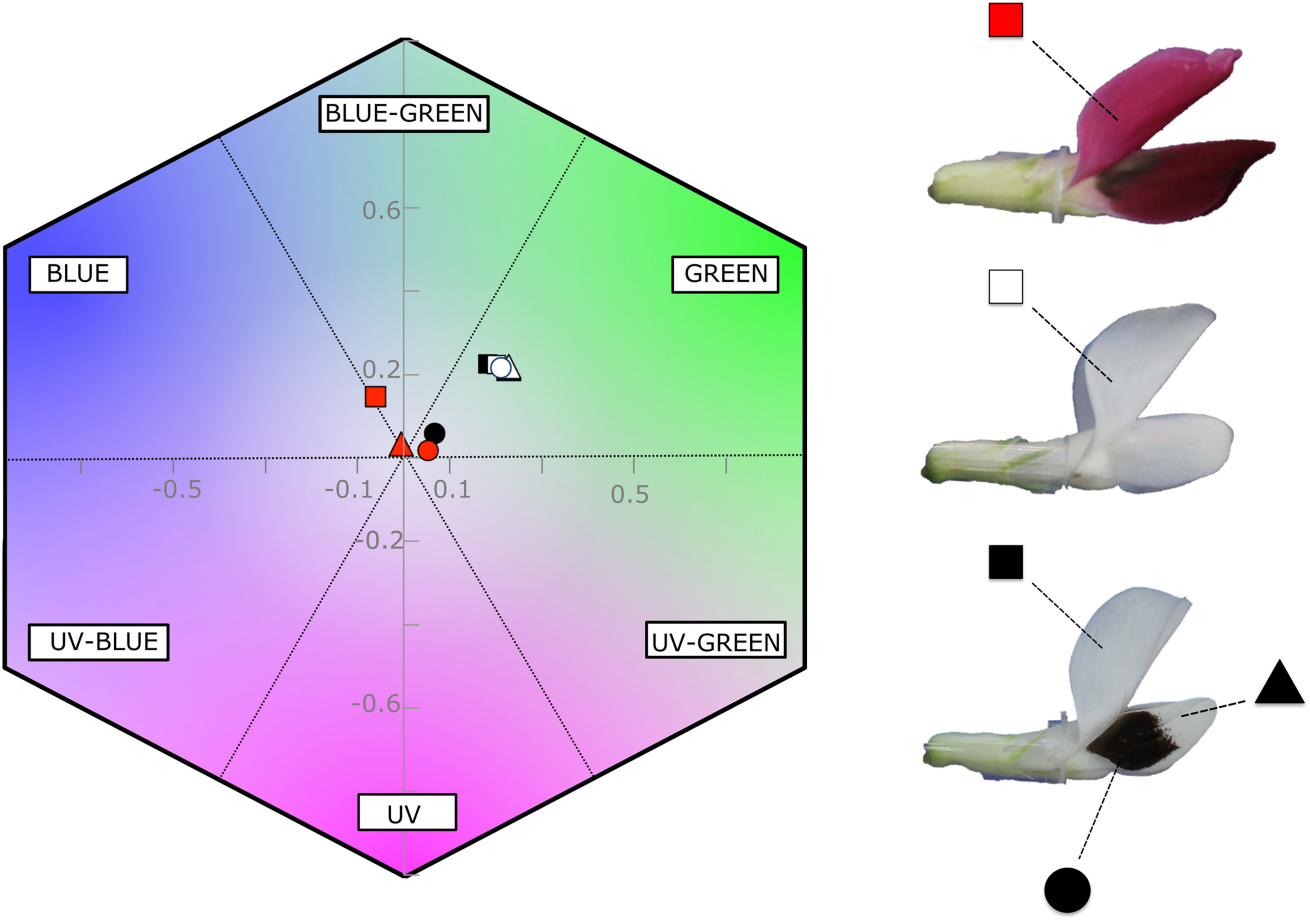
A summary of the major flower types of *Vicia faba* according to their colour in bee visual space. Shapes represent the location of the reflectance spectrum measured (Square = adaxial surface of standard, Triangle = tip of the abaxial surface of the wing, Circle = centre of the abaxial surface of the wing). Colours represent the three major flower types observed (Black = spotted flower type, Red = crimson flower type and White = non‐spotted type). Twenty‐five lines fell into the spotted type, four into the non‐spotted and one into the crimson type. Points are calculated as the mean blue, green and UV excitation values for the categories spotted, crimson and non‐spotted, averaged across the mean values for each line.

When plotted in bee colourspace (Chittka, 1992), three main types were apparent (Figure 2): ‘spotted’ (25 lines), ‘non‐spotted’ (4 lines) and ‘crimson’ (1 line). Standard petals and wing petal tips of both spotted and non‐spotted lines appeared blue‐green in bee colour space, whereas wing petal spots of spotted lines appeared more achromatic to bees (Figure 2). One line (NV706) was ‘crimson’, with bee‐blue‐coloured standard petals and achromatic wing petals. Only subtle variation in the colour of flowers was identified between spotted lines and between non‐spotted lines (Figures S5–S8 and Tables S9 and S10).

### 3.2 The size of the wing spot on *V. faba* flowers

**FIGURE 3:**
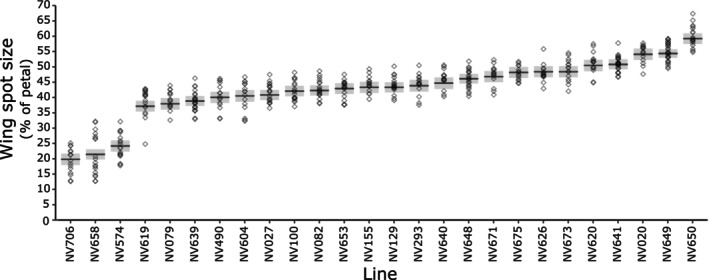
The wing petal spot size in spotted lines of *Vicia faba*. Model estimates for each line (black line) are plotted with 95% confidence intervals (grey bars). Raw data is represented by grey diamonds. Line was a significant predictor of the spot size (% of wing petal; *p* < .0001).

Within the lines with spots (‘spotted’ and ‘crimson’; Figure 2), a threefold difference in relative spot size was observed between the spotted lines of *V. faba* (Figure 3). Significant differences in spot size between lines were observed [*Line* likelihood ratio (25) = 471, *p* < .0001; Table 3], ranging from 20 [18, 22] % of the petal area (estimate [95% confidence interval]) in flowers of NV706 to 59 [57, 61] % in NV650, with a mean across lines of 43% (Figure 3).

**TABLE 3:** The significance of fixed effects in the best models.

Response variable	Likelihood ratio and significance of factors in best model		
	Line	Month	Replicate
%SpotArea	LR (25) = 471 *p* < .0001	LR(4) = 33.3 *p* < .0001	LR (2) = 22.3 *p* < .0001
StandardHeight	LR (28) = 341 *p* < .0001	LR(6) = 17.9 *p* = .006	NA
WgArea	LR (29) = 381 *p* < .0001	LR (6) = 53.5 *p* < .0001	LR (2) = 5.58 *p* = .061
Ln(WgShape)	LR (29) = 302 *p* < .0001	LR(6) = 15.1 *p* = .02	NA
Corolla‐tube length	LR (29) = 274, *p* < .0001	LR (6) = 27.0, *p* = .0001	NA

### 3.3 The size of standard petals in *V. faba*

**FIGURE 4:**
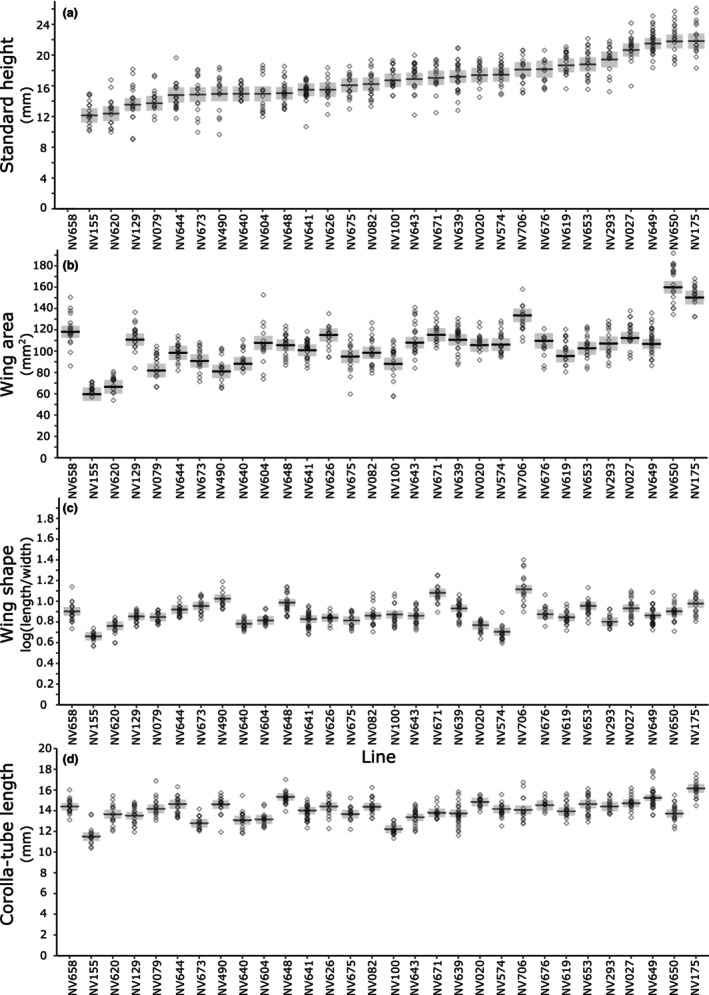
The morphology of *Vicia faba* flowers. (a) The height of the standard petal; (b) The area of a wing petal (c) The shape of wing petals (wing length: height ratio) (d) Corolla‐tube length. Model‐predicted means (black lines) with 95% confidence intervals (grey boxes) are plotted with the raw data (grey diamonds). Line was a significant predictor (*p* < .0001) of all four morphological measurements. The height of NV658 flowers was not calculated as this was a mutant with closed flowers.

There was a twofold difference in the absolute height of standard petals across the 29 lines measured, ranging from 12 [11, 13] mm in flowers of NV155 and NV620 (estimate [95% confidence interval]) to 22 [21, 23] mm in NV175 and NV650 (Figure 4a). This difference represents significant variation in the size of flowers between different genetic lines [*Line* likelihood ratio (28) = 341, *p* < .0001; Table 3].

### 3.4 The size of wing petals in *V. faba*

A 2.7‐fold difference in wing petal area was present between the *V. faba* lines measured in this study. Wing petal area ranged from 60 [53, 66] mm^2^ (estimate [95% confidence interval]) in NV155 to 160 [154, 166] mm^2^ in NV650, with an average wing petal area of 104 mm^2^ (Figure 4b). This difference represents significant variation between different lines [likelihood ratio (29) = 381, *p* < .0001; Table 3].

### 3.5 The shape of wing petals in *V. faba*

Wing shape varied 1.6‐fold between *V. faba* lines (after back‐transformation of estimates). Wing shape ratio varied significantly with line [likelihood ratio (29) = 302 and *p* < .0001; Table 3]; back‐transformed estimates ranged from 0.66 [0.62, 0.71] in line NV155 to 1.12 [1.08, 1.16] in NV706. On average, petals were 2.4 times as long as they were wide (Figure 4c).

### 3.6 The length of the corolla‐tube in *V. faba*

The corolla‐tube length of lines varied least, at 1.3‐fold, from 12 [11, 12] mm long in flowers of line NV155 and 12 [12, 13] mm in NV100 to 16 mm [16, 16] in NV175, compared with an average of 14 mm across all lines (Figure 4d). This difference represents significant variation in the length of corollas between different lines [likelihood ratio (29) = 274, *p* < .0001; Table 3].

### 3.7 Components of variance for morphological traits and spot size

To examine the importance of heritable factors in determining trait variation for flower morphology and wing spot size, components of variance were analysed. In every dataset, genotype (Line) explained the largest proportion of trait variation (corolla‐tube length 53%, wing shape 60%, standard height 66%, wing area 70% and wing spot size 87%), suggesting that these traits were heritable and the most important determinant of the trait variation that we measured. Although Month improved model fit and was a significant factor in all of the mixed linear models predicting flower traits (Table 3), suggesting some environmental influence on trait values, Month and Replicate explained a minor part of the floral trait variance (most often ≤2%; Table S8). Plant also explained a very small proportion of the variance (1%–4%), indicating that under glasshouse growing conditions, genotype rather than developmental or environmental factors are most important in determining flower traits.

### 3.8 The floral volatiles of *V. faba*

The total quantity of floral volatiles recovered from flowers of NV641 and NV676 ranged from 0.004 to 0.35 mg ocimene equivalents per sample, with a median of 0.088 mg ocimene equivalents produced per sample by NV641 and 0.059 by NV676. The total amount of volatiles collected did not differ significantly between lines (Mann–Whitney test, *W* = 15, *p* = .61), suggesting that the near absence of ocimene in NV676 does not result in a reduction in VOC production.

In total 15 VOCs were identified from *V. faba* flowers. The profile of these VOCs differed markedly between the two lines (Table 4 and Table S17 for absolute values). The monoterpenes, ocimene, linalool and tentatively limonene, myrtenol and an unidentified monoterpene, represented 86% of the volatiles collected from NV641 and 45% from NV676. This difference was largely due to the near absence of ocimene in NV676. Conversely, the sesquiterpenes, predicted to be caryophyllene and humulene, represented a much greater proportion of the volatiles in NV676 (40%) compared with NV641 (8%).

**TABLE 4:** The percentage of total VOCs by tentative identity of compound, obtained from flowers of *Vicia faba* lines NV641 and NV676.

Retention time (RT)	Predicted compound	% of volatiles produced (mean ± SE)		Number of extracts detected in	
		NV641	NV676	NV641	NV676
8.09	Ocimene^a^	69.6 (±3.6)	0.1 (±0.0)	6/6	1/4
8.76	Linalool^a^	14.1 (±2.4)	45.0 (±6.8)	6/6	4/4
11.92	Caryophyllene	6.3 (±1.7)	34.3 (±5.6)	5/6	4/4
12.45	Copaene/Germacrene	3.1 (±0.8)	6.3 (±2.2)	5/6	4/4
12.21	Humulene	1.6 (±0.3)	5.0 (±0.8)	6/6	4/4
10.92	Cinnamyl alcohol	0.5 (±0.5)	3.7 (±1.3)	1/6	3/4
12.79	Copanene/Napthalene	0.9 (±0.2)	2.5 (±0.6)	6/6	4/4
13.26/46	Caryophyllene oxide	0.7 (±0.3)	1.9 (±0.4)	5/6	4/4
7.95	Unidentified monoterpene	1.0 (±0.3)	0.0 (±0.0)	4/6	0/4
13.8	Cadinol/murrolol	0.1 (±0.1)	0.9 (±0.4)	2/6	4/4
12.66	Farnesene	0.7 (±0.2)	0.0 (±0.0)	5/6	0/4
10.57	Cinnamaldehyde	0.3 (±0.2)	0.3 (±0.2)	4/6	2/4
7.74	Limonene	0.4 (±0.4)	0.0 (±0.0)	1/6	0/4
9.26	Myrtenol	0.4 (±0.2)	0.0 (±0.0)	5/6	0/4
12.14	Cinnamyl acetate	0.2 (±0.1)	0.0 (±0.0)	3/6	0/4

*Note*: Shading indicates size of mean values (dark = largest, light = smallest). The predicted compounds for each peak based on their mass spectra are given. The compounds at the retention time of 12.45, 12.79 and 13.8 were each predicted to be one of two compounds with equal likelihood.

^a^ The identity of ocimene and linalool were confirmed using authentic standards.

### 3.9 Bee responses to variation in floral traits

#### 3.9.1 Experiment 1: Can bees perceive differences in the scent of flowers from NV641 and NV676?

**FIGURE 5:**
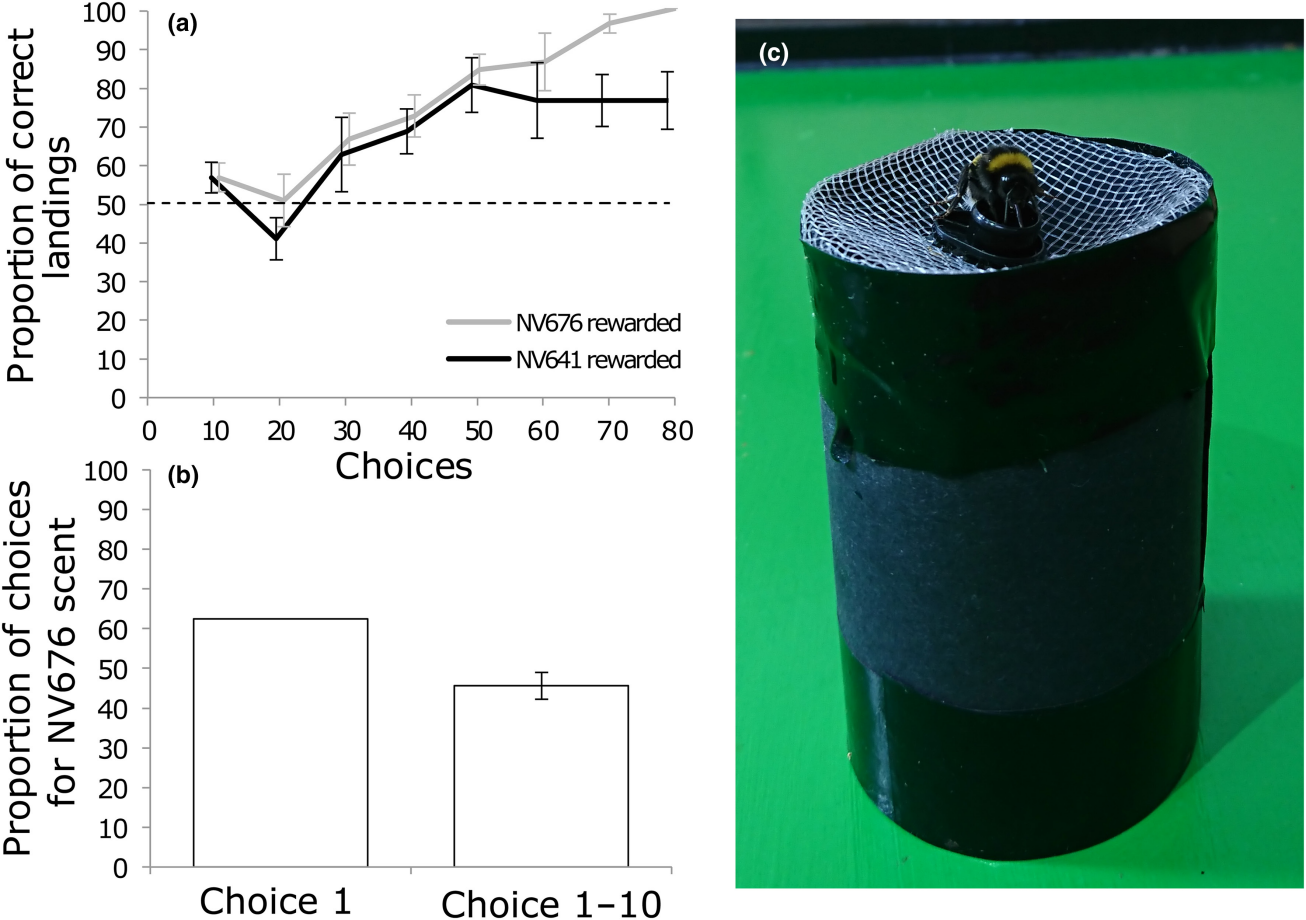
Bumblebee responses to the VOCs of NV641 and NV767. (a) The learning curve of *Bombus terrestris* foragers in a differential conditioning experiment where the scent of flowers from NV641 (black line, *n* = 5 foragers) or NV676 (grey line, *n* = 5 foragers) was rewarded. The unrewarded line was paired with a quinine distractor solution. The percentage of correct choices is plotted ±SE for 10 choice bins; the lines are offset for ease of viewing. There was a significant improvement in the proportion of correct choices over the course of the experiment for both lines, as well as an effect of rewarded scent (*p* < .001). (b) The percentage of 16 scent‐naïve foragers visiting NV676 scented towers on their first, and first 10, visits in a preference test where both scents were equally rewarded. The data are proportional and mean ± SE. There was no significant preference for either scent (*p* > .05). (c) A forager visits a scented tower during the preference experiment.

In the final 10 choices (71–80), the proportion of correct choices (rewarded towers) was 76% ± 8 (when associated with NV641) and 100% ± 0 (when associated with NV676) (mean ± SE). All foragers visited more than the 50% correct choices expected by chance. There was a significant interaction between the rate of learning (choice) and the scent that was rewarded during an experimental run [binomial logistic regression model; *χ* ^2^(1) = 11.3, *p* = .0008]. Regardless of which scent was rewarded, there was a significant improvement in the accuracy with which bees visited the rewarded scent [binomial logistic regression model for bees assigned the scent of NV676 as rewarding; *χ* ^2^(1) = 68.8; *p* < .0001; or of NV641 as rewarding; *χ* ^2^(1) = 19.7; *p* < .0001], indicating that *B. terrestris* foragers can perceive the difference in the scents of NV676 and NV641 flowers (Figure 5a).

#### 3.9.2 Experiment 2: Do bees prefer the scent of flowers from NV641 or NV676?

Despite being able to perceive a difference in scent (experiment 1), foragers had no strong preference between the scent of flowers from lines NV676 or NV641. On their first choice, 10/16 scent‐naïve bees landed on towers containing NV676 flowers (Figure 5b). Over their first 10 choices, foragers landed on a mean of 46% of towers containing NV676 flowers (Figure 5b). There was no significant difference between these values and those expected by chance [first choice: binomial test, *n* = 16, *p* = .45; first 10 choices: *t*(15) = −1.28, *p* = .22].

#### 3.9.3 Experiment 3: Do bees prefer flowers with wing petal spots?

**FIGURE 6:**
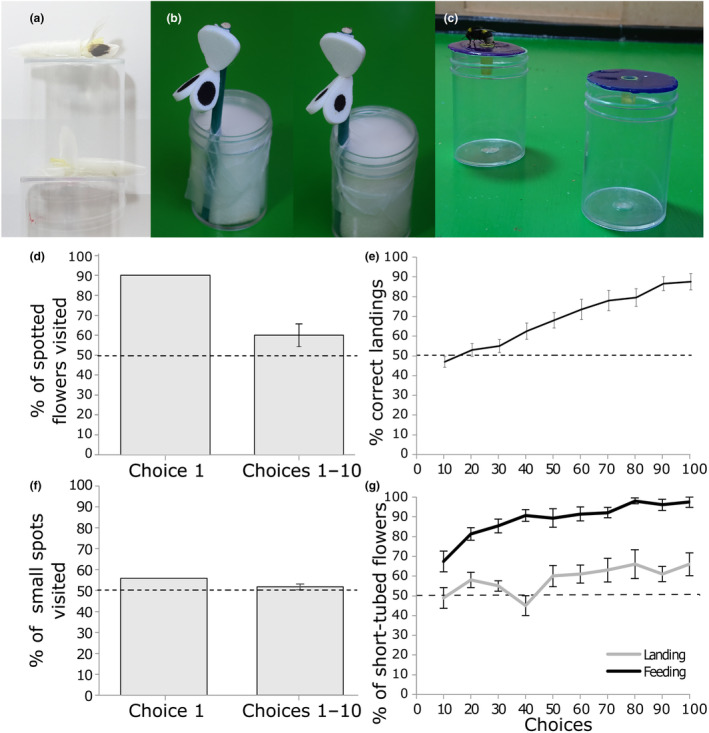
*Bombus terrestris* responses to spot size and corolla‐tube length. (a) The flowers used for spot preference experiments. Top: NV641 flower; Bottom: NV676 flower. (b) The flower models used for the spot size experiment. (c) The artificial flowers used for the tube‐length preference experiment (both are blue). (d) The preference for spotted NV641 over non‐spotted NV676 flowers in the foragers' (*n* = 10) (i) first and (ii) first 10 visits. Both flowers were equally rewarded. There was a significant preference for spotted flowers on the first choice (*p* < .05) but not over 10 choices (*p* > .05). (e) The choices of foragers between small and large spotted flowers over 100 choices. Correct choices were when a forager fed on the model rewarded with sugar; incorrect choices were paired with a quinine distractor solution (*n* = 20 bees, equally divided between large and small spotted model rewarded). There was a significant improvement in the proportion of correct choices over the course of the experiment (*p* < .0001). (f) The preference of foragers (*n* = 50) visiting large over small spotted flowers on their (i) first and (ii) first 10 visits, both flowers were equally rewarded. There was no significant preference between models at choice 1 or 10 (*p* > .05). (g) The preference of foragers between short‐tubed model flowers (12 mm long) vs. long‐tubed models (16 mm long) over 100 choices as they learned to associate tube length with flower colour. All foragers (*n* = 15) were recorded for 100 feeding events. For a subset of foragers (*n* = 10), their first 100 landings on a flower were recorded separately (grey line). There was a significant increase in the probability that a short‐tubed flower was chosen over the course of the experiment (landings; *p* < .01; feedings; *p* < .0001). In all subparts, error bars are SE. Means for 10 choice bins are plotted for (e, g) and (d, f) plot the proportion of first choices and the mean percentage preference over 10 choices.

Given that the flowers of NV676 and NV641 do not have significantly different morphologies (Tables S13–S16) and *B. terrestris* workers displayed no innate preference for the floral scent of either line (Figure 5), these lines were used to investigate whether *B. terrestris* has a preference for the wing petal spots of *V. faba* flowers. On their first choice, 9/10 scent‐naïve bees landed on spotted flowers (NV641) and one bee landed on non‐spotted flowers (NV676) (Figure 6d), suggesting that bees have a significant preference for spotted *V. faba* flowers (binomial test; *n* = 10, *p* = .021). Over the course of 10 choices, 60% of landings spotted flowers on average. This difference was not significantly different from random chance [*t*(9) = 1.73, *p* = .12].

#### 3.9.4 Experiment 4: Can bees perceive the difference between large and small spotted petal models?

To test whether bees can distinguish between different petal spot sizes, artificial flowers were made with spots equivalent to the largest and smallest percentage cover in our dataset (60% and 20%, respectively). In the final 10 choices (91–100) of the experiment, the proportion of correct choices was 88 ± 3%, and all foragers visited more than the chance expectation of 50% correct choices. There was a significant increase in the probability that the correct spot size was chosen over the course of the experiment [*χ* ^2^(1) = 107.4, *p* < .0001], demonstrating that *B. terrestris* can perceive differences in spot sizes in a model system (Figure 6e).

#### 3.9.5 Experiment 5: Do bees prefer large or small spotted wing petals?

Although foragers could perceive the differences in spot size at the extremes of our dataset, there was no innate preference between these. On their first choice, 28/50 bees landed on small, spotted model flowers (Figure 6f). Over the first 10 choices, 52% of bees landed on small, spotted flowers on average. These values were not significantly different from random choice [first choice: binomial test; *n* = 50, *p* = .021; first 10 choices: *t*(49) = 1.73, *p* = .12].

#### 3.9.6 Experiment 6: Do bees prefer flowers with shorter corolla tubes?

To determine whether bees preferred flowers with a corolla tube equivalent to the shortest (12 mm) or longest (16 mm) in our dataset, artificial flowers were made, with the corolla‐tube length paired with a colour cue (Figure 6c).

In the last 10 choices (91–100) of the experiment, the proportion of short‐tube discs landed on was 66 ± 6%. The proportion of short‐tubed discs landed on by foragers increased over the course of the experiment [*χ* ^2^(1) = 10.7, *p* = .0011], increasing from 48 ± 7% (mean ± SE) in the first 10 choices. This suggests a preference for landing on shorter‐tubed flowers, as bees have learned to associate this with the colour cue. There was also evidence that foragers preferred to land on purple flowers, with foragers assigned purple short‐tubed flowers visiting 74 ± 9% of these flowers at choices 91–100, compared with 58% ± 7% when short‐tubed flowers were blue [*χ* ^2^(1) = 5.1, *p* = .024]. There was no difference in the rate at which bees have learned to visit short‐tube flowers according to their colour assignment [choice: colour interaction, *χ* ^2^(1) = 0.6, *p* = .46].

Some bees were observed landing on flowers but not feeding from them. When analysing only the flowers fed from, 97 ± 3% short‐tubed flowers were fed from in the last 10 choices (91–100), compared with 67 ± 5% in the first 10 choices (Figure 6g). The proportion of short‐tubed flowers fed from it increased significantly over the duration of the experiment [*χ* ^2^(1) = 107.4, *p* < .0001]. There was no significant difference in the proportion of short‐tubed flowers fed according to their colour assignment [*χ* ^2^(1) = 0.14, *p* = .70].

## 4 DISCUSSION

Many important crop species are at least partially dependent on animal pollinators for yield (Klein et al., 2007). Therefore, identifying intraspecific floral trait variation that could improve the attractiveness of a flower to its pollinators is an important strategy for sustainably improving crop yield (Bailes et al., 2015; Palmer et al., 2009; Prasifka et al., 2018). Substantial differences have been found in the quantity of nectar and pollen produced in the crop *V. faba* and in petal epidermal morphology (Bailes et al., 2018; Bailes & Glover, 2018). We have identified similar genetically determined variation in ‘attractive’ traits of the same lines and show that bumblebees have preferences for, and can distinguish between, floral traits within the natural range of variation.

The extent of variation differed between traits measured in this study. Corolla‐tube length showed relatively small differences between the extremes observed (~1.4‐fold change between shortest and longest), whereas wing petal spot size varied greatly (~3 fold). Some floral traits may, therefore, be easier to manipulate through breeding than others. However, even small changes in floral characters can have measurable effects on pollinator preference, as demonstrated by our behavioural experiment, which indicated that bumblebees prefer shorter corolla tubes.

Based on our bee behavioural experiments, changes to corolla‐tube length may be one of the most useful traits to be selected for by breeders. In the field, shorter corolla tubes have been associated with greater crop visitation rates and outcrossing in sunflower and field bean (Mallinger & Prasifka, 2017; Portlas et al., 2018; Suso et al., 2005). Here, we show, under controlled conditions, that when the nectar reward is equal between ‘flowers’, shorter corolla tubes are still preferred by bumblebees. This is not surprising, as the corolla‐tube length is directly related to the accessibility of the floral reward—flower handing time decreases and sugar extraction rate increases with tongue length relative to corolla‐tube length (Inouye, 1980; Klumpers et al., 2019). The corolla‐tube length of *V. faba* is greater than the tongue length of most of its pollinators (6–7 mm in *Apis mellifera*, 7–8 mm in *B. terrestris/lucorum/lapidarius* and 12–13 mm in *B. hortorum*) (Alpatov, 1929; Goulson et al., 2005; Peat et al., 2005). The absolute difference in corolla‐tube lengths identified in this study (4 mm) is large relative to the tongue length of bumblebees. Indeed, studies of sunflowers in the field suggest a 1 mm change can make a substantial difference to visitation rates (Portlas et al., 2018). Although not possible to investigate in this study, pollinators with different tongue lengths are likely to respond differently to corolla‐tube length (Klumpers et al., 2019; Rojas‐Nossa et al., 2016). Therefore, future work should seek to explore preferences in different bee species, particularly the effect of tube length on the incidence of nectar robbing, which has been positively correlated to corolla‐tube length (Rojas‐Nossa et al., 2016) and can occur extensively in *V. faba* (e.g. Smith‐Ramirez et al., 2021). However, breeding for shorter corolla tubes is likely to be beneficial, given that in blueberry, shorter, wider corollas are associated with reduced nectar robbing (Courcelles et al., 2013).

We observed considerable variation in other aspects of floral morphology—the size of standard and wing petals, and wing petal shape. Larger flowers are more quickly located by bees (Tsujimoto & Ishii, 2017), and bees have often been shown to prefer large flowers, which typically contain a larger reward (Galen & Newport, 1987; Elle & Carney, 2003; Martin, 2004; Spaethe et al., 2001). However, mixed effects of flower dimensions on the rate of outcrossing have been reported in *V. faba* (Suso et al., 2005, 2008). These mixed results could be a result of pleiotropic effects leading to larger flowers being harder to open (Bailes et al., 2018; Córdoba & Cocucci, 2011; Córdoba et al., 2015) or changes in the zygomorphy of the flower. The influence of petal shape on plant–pollinator interactions is much less understood. In *Erysimum mediohispanicum* (Brassicaceae), bees preferred to visit flowers with narrower petals, but this is likely acting as a cue for a higher reward (Gómez et al., 2008). More work is needed before recommendations can be made to breeders on the optimal size and shape of flowers.

Previously, different colour phenotypes (as observable by humans) have been identified in *V. faba* (Duc, 1997; Hughes et al., 2020). However, as bees perceive different wavelengths, extending into the UV spectrum (Menzel & Blakers, 1976; Peitsch et al., 1992), there may be hidden variation with *V. faba*, such as the large variation in UV pigmentation of sunflowers (Todesco et al., 2022). In our study, only small‐colour contrast differences (~0.02 hexagon units) were observed within the three human‐observable types that were present within our panel. These differences may be perceivable by honeybees; however, it is unlikely that bumble bees are able to discriminate between subtle colour contrasts (Dyer et al., 2006, 2008; Dyer & Neumeyer, 2005). Therefore, we suggest specialist equipment is not needed when considering breeding for flower colour traits in *V. faba*.

Floral patterning (the presence of wing petal spots) is of particular importance in *V. faba* because non‐spotted lines are associated with low tannin seeds with greater utility for animal feed (Crépon et al., 2010). It has been proposed that petal spots can improve pollination by acting as nectar guides or by mimicking pollinating insects in some systems (Eisikowitch, 1980; Ellis et al., 2014; Goulson et al., 2009; Johnson & Dafni, 1998). Smaller spotted flowers of *Mimulus luteus* receive more bee visits than larger spotted flowers, but bee visitation may also be affected by spot shape and colour (Medel et al., 2003). We identified significant variation in wing petal spot size among ‘spotted’ *V. faba* lines. Our innate preference experiment suggests that naïve bumblebees have an initial preference for spotted flowers over non‐spotted flowers, but that quickly dissipates. Although bees did not show an innate preference between large and small spots, they were able to distinguish between them, as demonstrated by our conditioning experiment. Petal spots may be important in the *V. faba* system to signpost which petals a bee must alight on, as seen in *M. luteus* (Medel et al., 2003). Whilst our experiments under controlled conditions identified only an initial preference for spots, other benefits of nectar guides such as reduced handling time and likelihood of robbing have been reported (Leonard et al., 2013; Leonard & Papaj, 2011) and environmental factors such as wind can lead to context‐dependent benefits of floral traits (Alcorn et al., 2012). Therefore, further studies of this trait in the field would be valuable.

Scent has previously been linked to increased visitation rates in some crop systems (Klatt et al., 2013; Rodriguez‐Saona et al., 2011). Indeed, given that crops are grown *en masse*, long‐range attractive traits may be the most important in increasing visitation to a crop by allowing more foragers to locate the large food resource. Here, we identify differences in the scent of two *V. faba* lines that are detectable by bumblebees but for which they have no significant preference. Previous studies of *V. faba* floral volatiles have identified (E)‐ß‐ocimene, *(E)‐*caryophyllene and (R)‐linalool as the major components in differing proportions across six varieties (including ‘Maris Bead’, NV640) (Bruce et al., 2011; Griffiths et al., 1999; Hoffmeister & Junker, 2017; Sutton et al., 1992). The compounds identified in this study match those reported in previous studies of *V. faba* flowers. One of the major differences identified between the lines examined in this study was the near absence of ocimene and the greater quantities of linalool produced by the flowers of NV676. This represents a different mix of volatiles to those previously reported, with similar levels of ocimene to ‘Sutton Dwarf’ but a greater ratio of linalool: caryophyllene (Bruce et al., 2011). Electroantennography experiments by Henning and Teuber (1992) have previously suggested that honeybees have a preference for linalool, which has a similar structure to the honeybee Nasonov pheromone geraniol. In this study, no preference was found, perhaps because of differences in chemical communication in bumblebees (Granero et al., 2005). However, it is interesting to note that foragers appear to learn the scent of line NV676 more accurately after 80 choices when it is associated with a reward (100% correct choices) than when the scent of line NV641 is associated with a reward (76% correct choices). This asymmetric learning has been noted in a previous proboscis extension reflex experiment using differential conditioning to train bumblebees to the scent of linalool versus phenylacetaldehyde (Laloi & Pham‐Delègue, 2004). Therefore, although no distinct preference for the scent of NV676 was identified, it is possible that over a longer time period, a preference for NV676 may develop because the scent of the flower is learned more easily.

In this study, we have focused on examining isolated floral traits in controlled bumblebee behaviour experiments. This strategy has allowed us to separate out attractive traits that *B. terrestris* has innate preferences for, as opposed to traits that are correlated with floral reward in *V. faba*. However, future work will be needed to provide optimal guidance to breeders, considering the relative importance of these traits, and in particular to consider these traits in the more complex environment of the field. For example, due to technical constraints, we carried out our behavioural experiments using *B. terrestris*. However, in the field, pollinator communities vary depending on location (e.g. Aouar‐Sadli et al., 2008; Hutchinson et al., 2021; Pierre et al., 1999). Pollinator identity can impact floral trait preferences (e.g. Bauer et al., 2017; Erickson et al., 2022). Therefore, whilst *B. terrestris* is often a numerous pollinator to *V. faba*, the preferences of other more efficient pollinators, such as long‐tongued *Bombus hortorum*, could be important in some communities (Marzinzig et al., 2018). In addition, different suites of floral traits may impact bee preference and learning. For example, some traits or trait combinations may be more salient than others. Bees learn which flowers are rewarding more quickly when a colour or shape cue is paired with a scent or when a colour is paired with a shape cue (Austin et al., 2019; Kulahci et al., 2008; Kunze & Gumbert, 2001; Leonard et al., 2011). In fact, whilst we have focused on traits attractive regardless of floral reward, novel traits can be rapidly learned as associated with a floral reward (e.g. Iridescence—Moyroud et al., 2017). Therefore, pairing traits such as the scent of NV676, which was rapidly and accurately learned by bees, with enhanced rewards that have already been identified in the *V. faba* gene pool (Bailes et al., 2018), may also be a promising strategy for breeders.

## 5 CONCLUSIONS

Our study has identified considerable variation in key floral traits that may function to attract pollinators and show promise as breeding targets. Lines of *V. faba* show substantial variation in flower dimensions and floral volatile emissions. Of the traits examined, spot presence appears to affect pollinator behaviour, with preference for spotted flowers over non‐spotted. Although variation in traits, including petal spot size and floral scent, does not appear to stimulate innate preference in bees, foragers can quickly learn to associate novel cues with a reward. Extremes of trait variation may therefore provide useful cues for bees when paired with enhanced reward. Floral traits associated with accessibility, including corolla‐tube length and visibility, including wing petal spot patterning, may be promising traits for breeders. Future work should seek to compare pollinator preference in the field when presented with *V. faba* lines displaying extremes of trait variation and should also explore the effect of additional attractive traits on bee behaviour, including floral colour and standard petal patterning.

## AUTHOR CONTRIBUTIONS

**Emily J. Bailes:** Conceptualization (lead); data curation (lead); formal analysis (lead); investigation (lead); methodology (lead); validation (lead); visualization (lead); writing – original draft (lead); writing – review and editing (equal). **Jake Moscrop:** Investigation (equal); writing – original draft (equal); writing – review and editing (equal). **Sarah Mitchell:** Investigation (supporting); writing – review and editing (equal). **Matthew Dorling:** Investigation (supporting); writing – review and editing (equal). **Tom Wood:** Conceptualization (equal); supervision (equal); writing – review and editing (equal). **Jane Thomas:** Conceptualization (equal); supervision (equal); writing – review and editing (equal). **Beverley J. Glover:** Conceptualization (equal); funding acquisition (equal); methodology (equal); resources (equal); supervision (equal); writing – original draft (equal); writing – review and editing (equal).

## CONFLICT OF INTEREST STATEMENT

The authors have no conflicts of interest.

## Supporting information


Data S1:

## Data Availability

The raw data generated for this paper, and the associated R code for analysis of data are available at: https://doi.org/10.17863/CAM.101998.
