# Supplementary material for: Bumblebee responses to variation in pollinator‐attracting traits of Vicia faba flowers
Source: Ecol Evol. 2023 Nov 10;13(11):e10617. doi: 10.1002/ece3.10617 (PMC10638492; doi:10.1002/ece3.10617)
Supplement: Supplementary file 1 — Data S1: [file ECE3-13-e10617-s001.docx]

# Supplementary methods

**Table S1 -** The seed source for the lines used in this study. Information supplied by the National Institute of Agricultural Botany. Lines were picked at random with the exception of those indicated with a * which were selected based on known colour variation or being commercial derivatives.

| **Line** | **Seed source** | **Donor ref and other names** | **Origin** |
| --- | --- | --- | --- |
|  |  |  |  |
| NV020 | ICARDA | ig11290 | landrace |
| NV027 | ICARDA | ig11312 | landrace |
| NV079 | ICARDA | ig11687 | landrace |
| NV082 | ICARDA | ig11695 | landrace |
| NV100 | ICARDA | ig11749 | landrace |
| NV129 | ICARDA | ig12137 | landrace |
| NV155 | ICARDA | ig12684 | landrace |
| NV175 | ICARDA | ig13004 | landrace |
| NV293 | ICARDA | ig70726 | landrace |
| NV490 | ICARDA | ig124213 | landrace |
| NV574 | ICARDA | ig130638 | landrace |
| NV604 | GRU | V185, Borington Bulk | landrace |
| NV619 | NICK-ADV (Limagrain UK) | NA12,  LAN08935 | breeding line |
| NV620 | CSIC | CSIC,  Vf172 | landrace |
| NV626 | CBP-T  (KWS) | KWS6,  NPZ 7-7301 | breeding line |
| NV639* | GOTTINGEN | 70176/70175, Hedin | commercial variety |
| NV640* | WA Church | Maris Bead | commercial variety |
| NV641* | LSPB | Fuego | commercial variety |
| NV643* | Plant Breeding Station, Strzelce | Albus | commercial variety |
| NV644* | Plant Breeding Station, Strzelce | Kasztelan | commercial variety |
| NV648* | ICARDA | ig101769, BPL10 | landrace |
| NV649 | ICARDA | ig101770, BPL11 | landrace |
| NV650 | ICARDA | ig101771, BPL12 | landrace |
| NV653 | ICARDA | ig101786, BPL27 | landrace |
| NV658* | GOTTINGEN | CGN07715 cf-3 (60354-9), closed flower mutant | landrace |
| NV671 | PBI | Atlas | commercial variety |
| NV673 | LSPB | Fury | commercial variety |
| NV675 | Limagrain UK | Pyramid | commercial variety |
| NV676* | LSPB | Tattoo | commercial variety |
| NV706* | Thompson & Morgan | Broad Bean Crimson Flowered | horticultural variety |

**Table S2** - Level of replication for flower morphology and spot size measurements Three flowers were measured from each plant. Lines that were excluded from the analyses using StandardHeight and %SpotArea are indicated with ^a^ and ^b^ respectively.

| **Line** | **N plants** |
| --- | --- |
| NV020 | 5 |
| NV027 | 6 |
| NV079 | 5 |
| NV082 | 6 |
| NV100 | 6 |
| NV129 | 6 |
| NV155 | 5 |
| NV175^b^ | 5 |
| NV293 | 5 |
| NV490 | 5 |
| NV574 | 6 |
| NV604 | 5 |
| NV619 | 6 |
| NV620 | 5 |
| NV626 | 5 |
| NV639 | 8 |
| NV640 | 5 |
| NV641 | 9 |
| NV643^b^ | 7 |
| NV644^b^ | 6 |
| NV648 | 7 |
| NV649 | 9 |
| NV650 | 6 |
| NV653 | 6 |
| NV658^a^ | 6 |
| NV671 | 5 |
| NV673 | 5 |
| NV675 | 5 |
| NV676^b^ | 4 |
| NV706 | 5 |


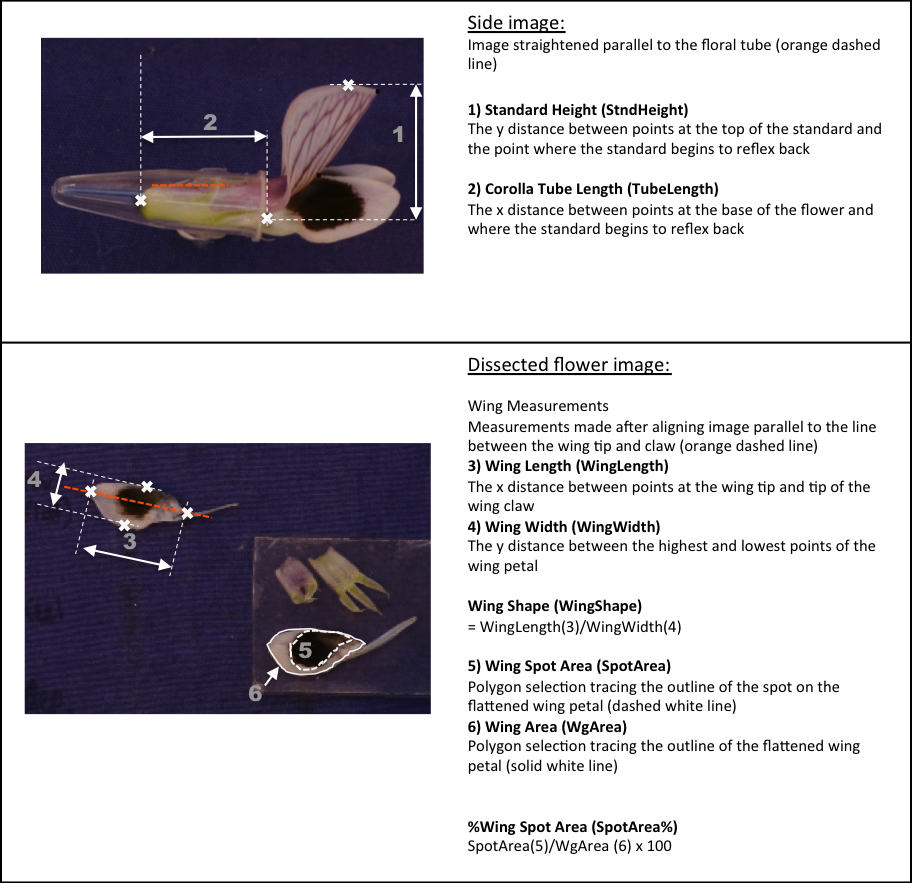


**Figure S1 –** Measurements made on photographed flowers

**Figure S2 –** The proportion of flowers measured in each month for each line.

## Volatile Organic Compound Analysis

### Air entrainment

The volatile organic compounds (VOCs) of lines NV641 and NV676 were analysed for differences in their composition using headspace analysis. Five dynamic headspace collections were made from flowers of each line following a method adapted from Beale et al. (2006). Each collection involved removing 8 open flowers (stage 4 or 5) from 2 – 3 plants of the same line, then placing them in a Pyrex glass container filled with distilled water. Plants were not re-used between replicates. The flowers were then sealed in a cylindrical glass collecting vessel, with a diameter of 8.5cm and a height of 7cm, using a metal base plate secured by bulldog clips and PTFE tape (Figure S2). All glassware and the metal plates had been washed in 0.2% Teepol to remove organic residues, followed by 100% acetone to remove ionic residues, then distilled water and baked at 180°C for 2 hours to degrade any remaining volatiles before use. Air was then passed through an activated charcoal-filter before entering the bottom of the chamber containing the flowers at a rate of 800ml/min. Air was removed from the chamber at a rate of 700ml/min through a Propak Q filter (50mg, 50/80 mesh size, Supelco (Sigma-Aldrich)) contained inside a glass gas chromatograph inlet liner between two plugs of silanised glass wool, preventing contamination of the air from that outside the chamber. Headspace collection was carried out for 24 hours. The entrained VOCs were then eluted from the column using 700µl of diethyl ether. Samples were stored at -80°C. The column was then washed with 1ml of diethyl ether three times before reuse; immediately after eluting the sample, 1 hour later, and 24 hours after that. The column was left for 24 hours for residual diethyl ether to evaporate before reuse. Along with the volatiles from the flowers of *V. faba,* two positive controls and two negative controls were collected for analysis. The positive controls were collected as for experimental samples, using eight flowers, but were spiked with an additional 2µl of either α-pinene or omiciene (66% & 29%, of trans and cis isomers respectively) standards before being enclosed in the collection vessel. The positive controls ensured that volatile collection and the subsequent GCMS analysis was working efficiently and that compounds could be identified correctly. The negative control was carried out as for experimental samples except that flowers were not added to the glass pyrex containers of water. This control was used to check for background noise during GCMS and control for any contamination of samples during their collection.

#### Coupled Gas Chromatography-Mass Spectrometry

To determine the volatile organic compounds produced by flowers of *V. faba,* a Thermo Scientific TRACE 1310 Gas Chromatograph with a capillary GC column (Zebron ZB-1 50 m x 0.32mm I.D., 0.5µm film thickness, Phenomenex) coupled with a Thermo Scientific ISQ™ LT Single Quadrupole Mass Spectrometer was used to analyse samples. The oven temperature was maintained at 30 ºC for 5min then programmed to increase the temperature to 230°C at 15°Cmin^-1^. Total run time was 18.33min. 1µl of sample was injected into the column using a splitless injection. The inlet temperature was set at 200ºC, the MS transfer line 240ºC, and the ion source temperature 250ºC. Ionisation was by electron impact (EI). A constant flow carrier gas of helium was used at a flow rate of 2.6 ml/min. A split flow of 26ml/min was used and a splitless time of 0.8min. MS mass range was 30-650 m/z with a dwell time of 0.35 sec. Compounds were identified by comparing their mass spectra with mass spectra databases (National Institute of Standards and Technology, 2005). The isomeric composition of compounds was not investigated.


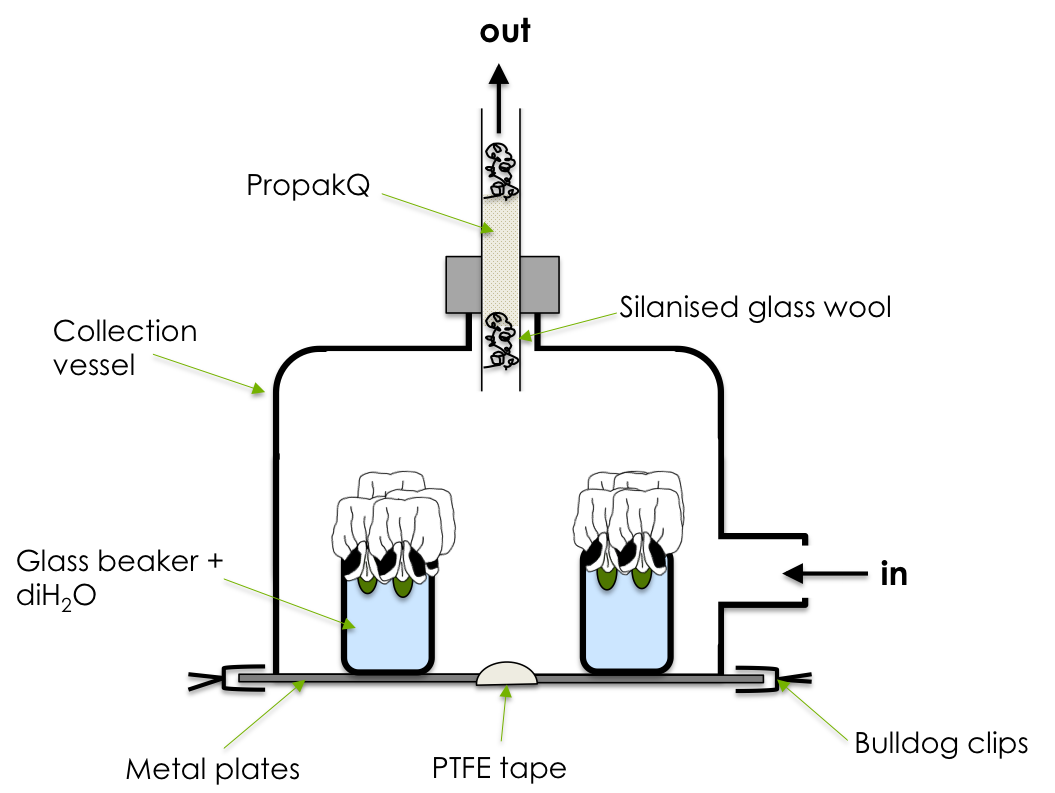


**Figure S3** – Setup for dynamic headspace collection of floral volatiles. Charcoal filtered air was pumped into the glass collection vessel at the location marked ‘in’, air was drawn out over eight open flowers, contained in 10ml glass beakers of deionized water, through a Propak Q filter at the location marked ‘out’. Black arrows indicate direction of airflow. The collection vessel was sealed using bulldog clips to seal the base with metal plates, and PTFE to seal the hole in the plates usually used to insert a plant stem through.

#### Calculating the quantity of volatiles produced by flowers

The quantity of volatiles produced by a flower, was calculated in mg ocimene equivalents using a standard curve of ocimene. Ocimene (Sigma Aldrich, mixture of isomers ≥ 90% purity) was diluted using diethyl ether (Sigma Aldrich, > 99.9%) to produce solutions of 2mg/ml, 1mg/ml, 0.5mg/ml, 0.25mg/ml, 0.125mg/ml, 0.0625mg/ml, and 0.03125mg/ml. Samples were then analysed by GCMS following 0. Following this, the area under the peaks identified as ocimene were measured, and plotted against the concentration of the solution. This allowed the concentration of the compounds in floral volatile samples to be estimated from their peak area.

## Bee experiments: creation of artificial flowers

### Design of tower-feeders for volatile experiments

Tower-feeders (Figure S3) were used to eliminate all other floral cues with the exception of volatile organic compounds. These were created by surrounding an ARACON with black card to produce a removable, 8.5 cm high, cylinder secured with double-sided sticky-tape. The top and bottom of the cylinder were reinforced with black electrical tape 2 cm thick that allowed these surfaces to be wipe-clean to remove scent marks and also secured 2 mm^2^ mesh. At the center of the ARACON there was a black 1.5 ml tube secured with PTFE tape, which was used as a vessel to contain flowers of *Vicia faba* in water during experiments. On the top of the tower, the lid of a black 1.5 ml tube was affixed using epoxy resin. This prevented UV reflectance of the quinine solution from being visible to bees when used as a distractor solution.

Figure S4 – Design of Tower-feeders for volatile experiments

#### Design of spot size models

To investigate the effect of wing petal spot size on bee preference, two sets of epoxy models were made, representing extremes of variation in *V. faba* wing spots (20% and 50% of wing area). Dental silicone (Zhermack elite HD+ dental silicone, Italy) moulds were made of the adaxial surface of white rose petals to mimic the conical cell surface. Conical cell structure of rose petals was confirmed using cryo- SEM prior to creating moulds. Casts were produced from moulds using Devcon epoxy resin coloured with Titanium white Artist’s pigment (Cornelissen & Son). Wing spots were marked onto model wing petals using a black permanent marker and a template. Two sets of models were made, one with spots covering 60% of the wing petal area and one covering 20% of the wing area, representing the variation observed in real *V. faba* flowers. Casts were mounted on dowels supported in Hamilton jars.

#### Generation of artificial disks for use in tube length experiments

Dental silicone (Zhermack elite HD+ dental silicone, Italy) casts were made of the lid of a 60 ml Hamilton jar to produce a round mould with an indent at its centre. These moulds were then filled with epoxy-resin (ITW Devcon, USA) mixed with powdered pigment (Purple or blue; Cornelissen & Son). A hole was made in the center of each disk, into which a 200 µl pipette tip (cut-down to 12, 14 or 16mm) was added to the center and sealed with parafilm at the base.

# Supplementary results

# The morphology of V. faba flowers

### Standard height

**Table S3 –** The AICc values for the models of standard height. Model average results include all models shaded in grey.

| **Line** | **Month** | **Rep** | **df** | **logLik** | **AICc** | **delta** | **weight** |
| --- | --- | --- | --- | --- | --- | --- | --- |
| + | + | na | 37 | -991.71 | 2063.47 | 0 | 0.7 |
| + | + | + | 39 | -990.75 | 2066.24 | 2.77 | 0.18 |
| + | na | na | 31 | -1000.68 | 2067.56 | 4.09 | 0.09 |
| + | na | + | 33 | -999.38 | 2069.54 | 6.07 | 0.03 |
| na | + | na | 9 | -1162.23 | 2342.82 | 279.34 | 0 |
| na | + | + | 11 | -1161.55 | 2345.64 | 282.16 | 0 |
| na | na | + | 5 | -1173.3 | 2356.72 | 293.25 | 0 |
| na | na | na | 2 | -1479 | 2962.02 | 898.55 | 0 |

The predictions of standard height for each line from the best model (including Line and Month only) were highly correlated with the predictions from model averaging (r = 0.99995). The model averaging approach predicts line NV155 to have the smallest flowers at 12 [11, 13] mm, NV650 and NV175 to have the largest flowers 22 [21,23] and an average flower height of 17 mm. These values are identical to those of the best model.

### Wing spot percentage cover

**Table S4 –** The AICc values for the models of spot percentage

| **Line** | **Month** | **Rep** | **df** | **logLik** | **AICc** | **delta** | **weight** |
| --- | --- | --- | --- | --- | --- | --- | --- |
| + | + | + | 36 | -1180.48 | 2439.32 | 0.00 | 1.00 |
| + | + | na | 34 | -1191.65 | 2456.96 | 17.63 | 0 |
| + | na | + | 30 | -1197.11 | 2458.60 | 19.27 | 0 |
| + | na | na | 28 | -1205.16 | 2470.12 | 30.79 | 0 |
| na | + | + | 11 | -1416.01 | 2854.62 | 415.29 | 0 |
| na | na | + | 5 | -1423.46 | 2857.06 | 417.74 | 0 |
| na | + | na | 9 | -1425.45 | 2869.31 | 429.99 | 0 |
| na | na | na | 2 | -1663.63 | 3331.28 | 891.95 | 0 |

There was no need to average the models for wing spot percentage as the best model, containing all three explanatory variables gave the best predictions by a large margin.

### Corolla tube length

**Table S5 –** The AICc values for the models of corolla tube length

| **Line** | **Month** | **Rep** | **df** | **logLik** | **AICc** | **delta** | **weight** |
| --- | --- | --- | --- | --- | --- | --- | --- |
| + | + | na | 38 | -606.74 | 1295.63 | 0.00 | 0.72 |
| + | + | + | 40 | -605.36 | 1297.55 | 1.92 | 0.28 |
| + | na | na | 32 | -620.22 | 1308.78 | 13.15 | 0 |
| + | na | + | 34 | -619.20 | 1311.30 | 15.67 | 0 |
| na | na | + | 5 | -745.48 | 1501.07 | 205.44 | 0 |
| na | + | na | 9 | -743.73 | 1505.81 | 210.18 | 0 |
| na | + | + | 11 | -742.60 | 1507.72 | 212.09 | 0 |
| na | na | na | 2 | -1208.18 | 2420.38 | 1124.75 | 0 |
|  |  |  |  |  |  |  |  |

The predictions of corolla tube length for each line from the best model (including Line and Month only) were highly correlated with the predictions from model averaging (r = 0.9999969). The model averaging approach predicts lines NV155 and NV100 have the shortest corolla tube at 12 [11, 12] mm and 12 [12,13] mm, NV175 to have the longest corolla tube length at 16 mm [16,16] and an average corolla tube length of 14 mm. These values are identical to those of the best model.

### Wing area

**Table S6 –** The AICc values for the models of wing area

| **Line** | **Month** | **Rep** | **df** | **logLik** | **AICc** | **delta** | **weight** |
| --- | --- | --- | --- | --- | --- | --- | --- |
| + | + | + | 40 | -1997.95 | 4082.72 | 0.00 | 0.61 |
| + | + | na | 38 | -2000.74 | 4083.62 | 0.90 | 0.39 |
| + | na | + | 34 | -2024.70 | 4122.28 | 39.56 | 0 |
| + | na | na | 32 | -2027.29 | 4122.89 | 40.17 | 0 |
| na | + | + | 11 | -2188.45 | 4399.42 | 316.70 | 0 |
| na | na | + | 5 | -2195.46 | 4401.03 | 318.30 | 0 |
| na | + | na | 9 | -2191.91 | 4402.16 | 319.44 | 0 |
| na | na | na | 2 | -2489.83 | 4983.68 | 900.95 | 0 |

The predictions of wing area (mm^2^) for each line from the best model (including Line, Month and Replicate) were highly correlated with the predictions from model averaging (r = 0.9999973). The model averaging approach predicts line NV155 to have the smallest wing petal at 60 [53, 66] mm^2^, NV650 to have the longest corolla tube length at 160 mm^2^ [154,166] and an average corolla tube length of 104 mm^2^. These values are identical to those of the best model.

**Table S7 –** The AICc values for the models of wing shape

| **Line** | **Month** | **Rep** | **df** | **logLik** | **AICc** | **delta** | **weight** |
| --- | --- | --- | --- | --- | --- | --- | --- |
| + | + | na | 38 | 602.90 | -1123.66 | 0.00 | 0.45 |
| + | na | na | 32 | 595.35 | -1122.39 | 1.27 | 0.24 |
| + | + | + | 40 | 604.41 | -1122.00 | 1.66 | 0.19 |
| + | na | + | 34 | 597.00 | -1121.12 | 2.54 | 0.12 |
| na | na | + | 5 | 448.00 | -885.89 | 237.77 | 0 |
| na | + | na | 9 | 452.11 | -885.87 | 237.78 | 0 |
| na | + | + | 11 | 453.01 | -883.50 | 240.16 | 0 |
| na | na | na | 2 | 77.14 | -150.26 | 973.40 | 0 |

The predictions of wing area (mm^2^) for each line from the best model (including Line, Month and Replicate) were highly correlated with the predictions from model averaging (r = 0.999473). Back transformed estimates from our model predict the widest petals to be in line NV155 at 1.9 [1.9, 2.0], and narrowest in NV706 at a ratio of 3.1 [3.0, 3.1]. On average petals were 2.4 times as long as they were wide. These figures are almost identical to those of the best model, with NV706 predicted at 3.1 [3.0,3.2] in the best model.

### Variance components analysis

**Table S8** – The percentage of variance explained by each factor within the final models. Variance explained by “Line” represents genetic variation between lines, and the remainder of the variance components represent environmental variation, developmental variation and measurement error. Percentages do not add to 100% because of residual variance not explained by the factors below. Residual variance will also represent environmental variation, developmental variation and measurement error.

| **Trait measured** | **Variance components** | | | |
| --- | --- | --- | --- | --- |
|  | **Line** | **Plant** | **Month** | **Replicate** |
| **Wing-spot size** | 87 % | 1 % | 1 % | < 1 % |
| **Standard height** | 66 % | 2 % | 1 % | NA |
| **Wing area** | 70 % | 4 % | 7 % | NA |
| **Wing shape** | 60 % | 2 % | 1 % | NA |
| **Tube-length** | 53 % | 3 % | 2 % | NA |

The colour of V. faba flowers


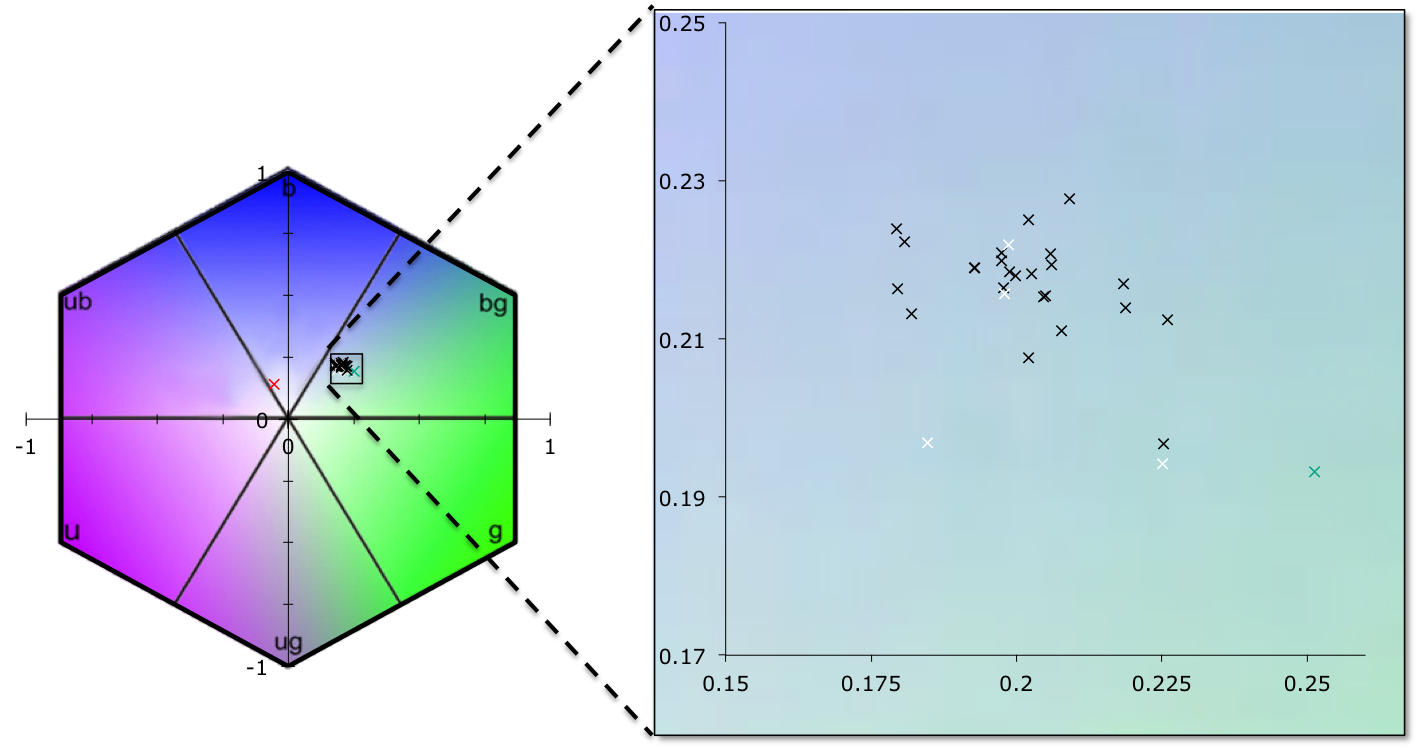


**Figure S5 -** The colour of the standard in bee-colour space of *V. faba* lines. Left – an overview of the bee-colour of all 30 lines of *V. faba* measured. Right – a close-up of bee-colour space showing the colour of the standard in spotted and non-spotted lines. Crimson flowered line NV706 is coloured red, non-spotted lines white, and spotted lines black, with the exception of the closed flower mutant NV658 that is coloured green. Each point represents the average of measurements from a minimum of 10 flowers.


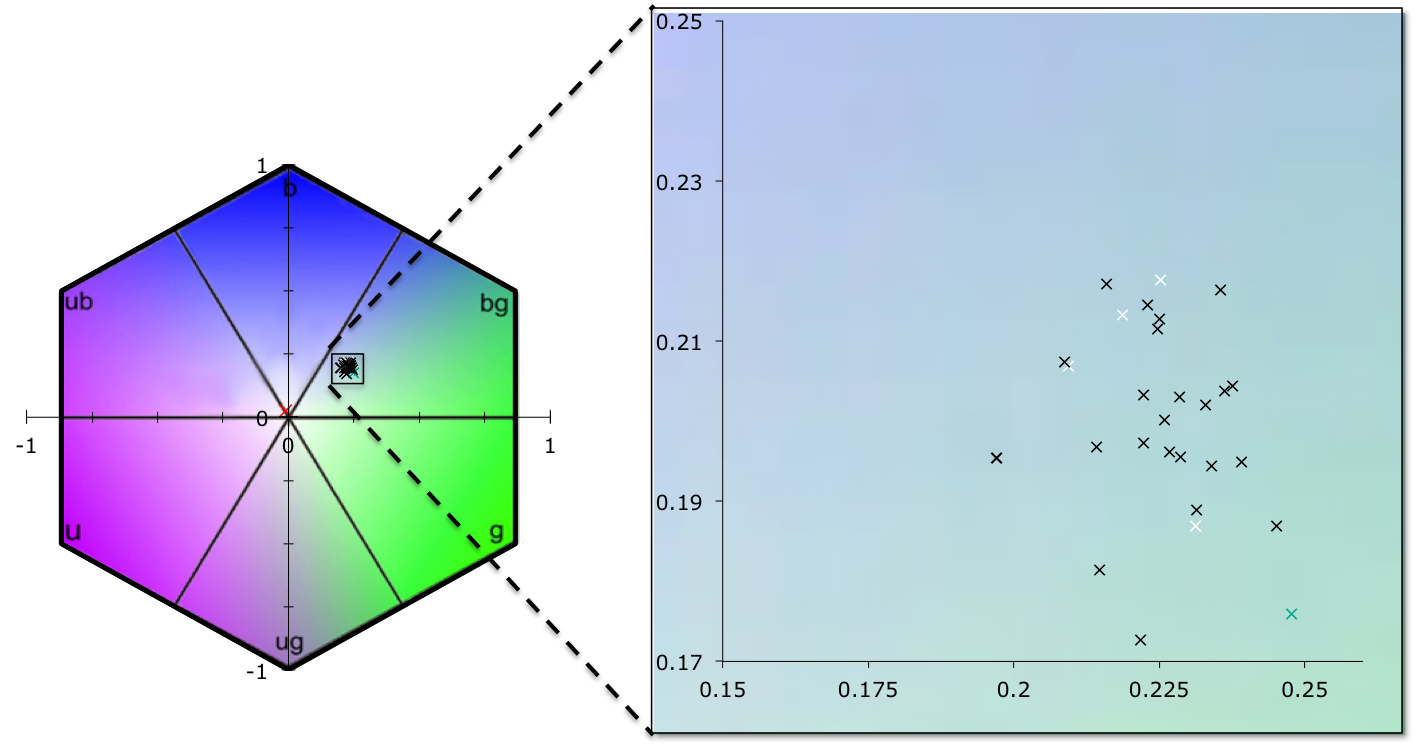


**Figure S6** - The colour of the wing tip in bee-colour space of *V. faba* lines. Left – an overview of the bee-colour of all 30 lines of *V. faba* measured. Right – a close-up of bee-colour space showing the colour of the wing tip in spotted and non-spotted lines. Crimson flowered line NV706 is coloured red, non-spotted lines white, and spotted lines black, with the exception of the closed flower mutant NV658 that is coloured green. Each point represents the average of measurements from a minimum of 10 flowers.


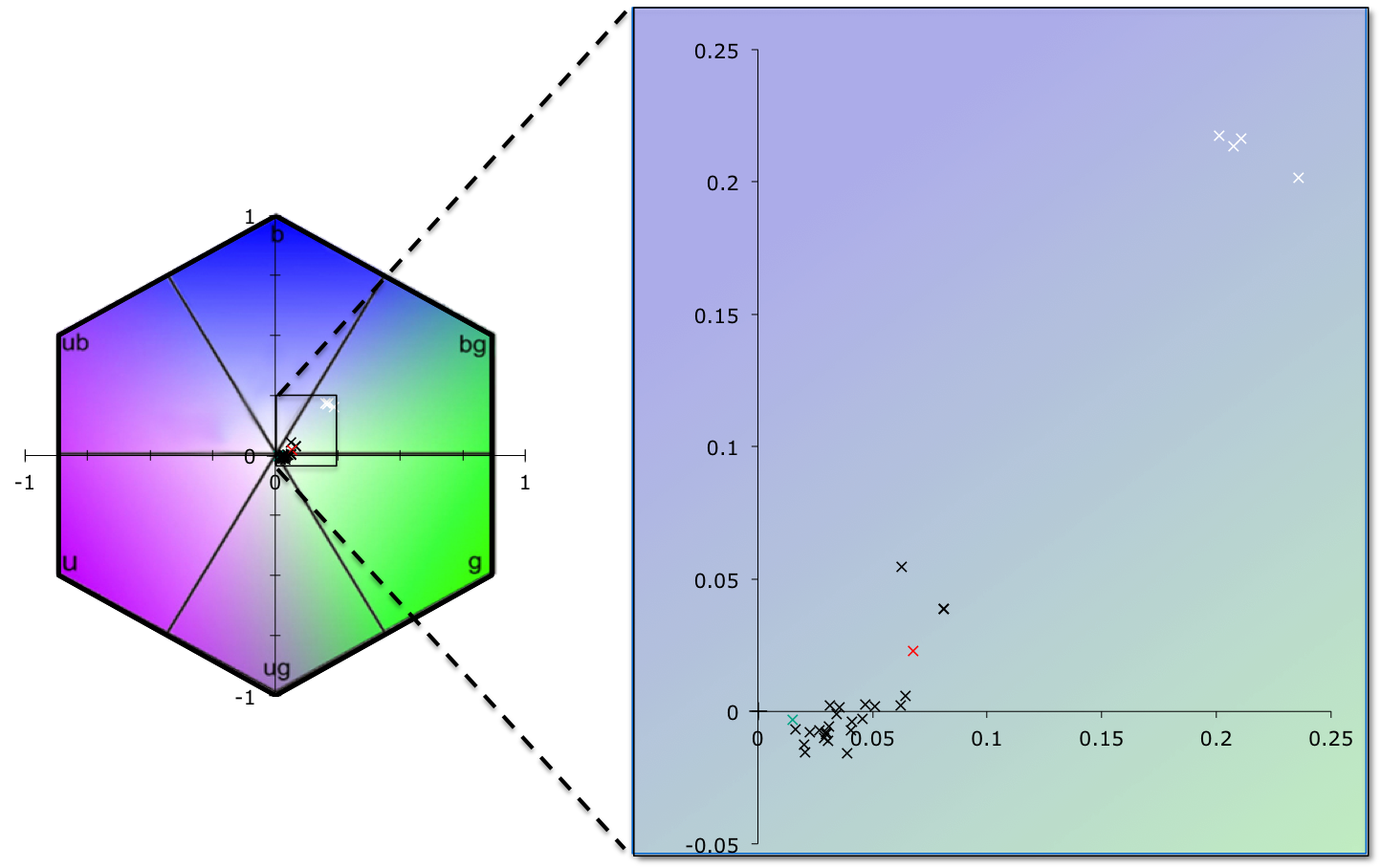


**Figure S7 -** The colour of *V. faba* lines in bee-colour space at the center of the wing petal where the wing spot is located. Left – an overview of the bee-colour of all 30 lines of *V. faba* measured. Right – a close-up of bee-colour space showing the colour of the wing spot in more detail. Crimson flowered line NV706 is coloured red, non-spotted lines white, and spotted lines black, with the exception of the closed flower mutant NV658 that is coloured green. Each point represents the average of measurements from a minimum of 10 flowers.


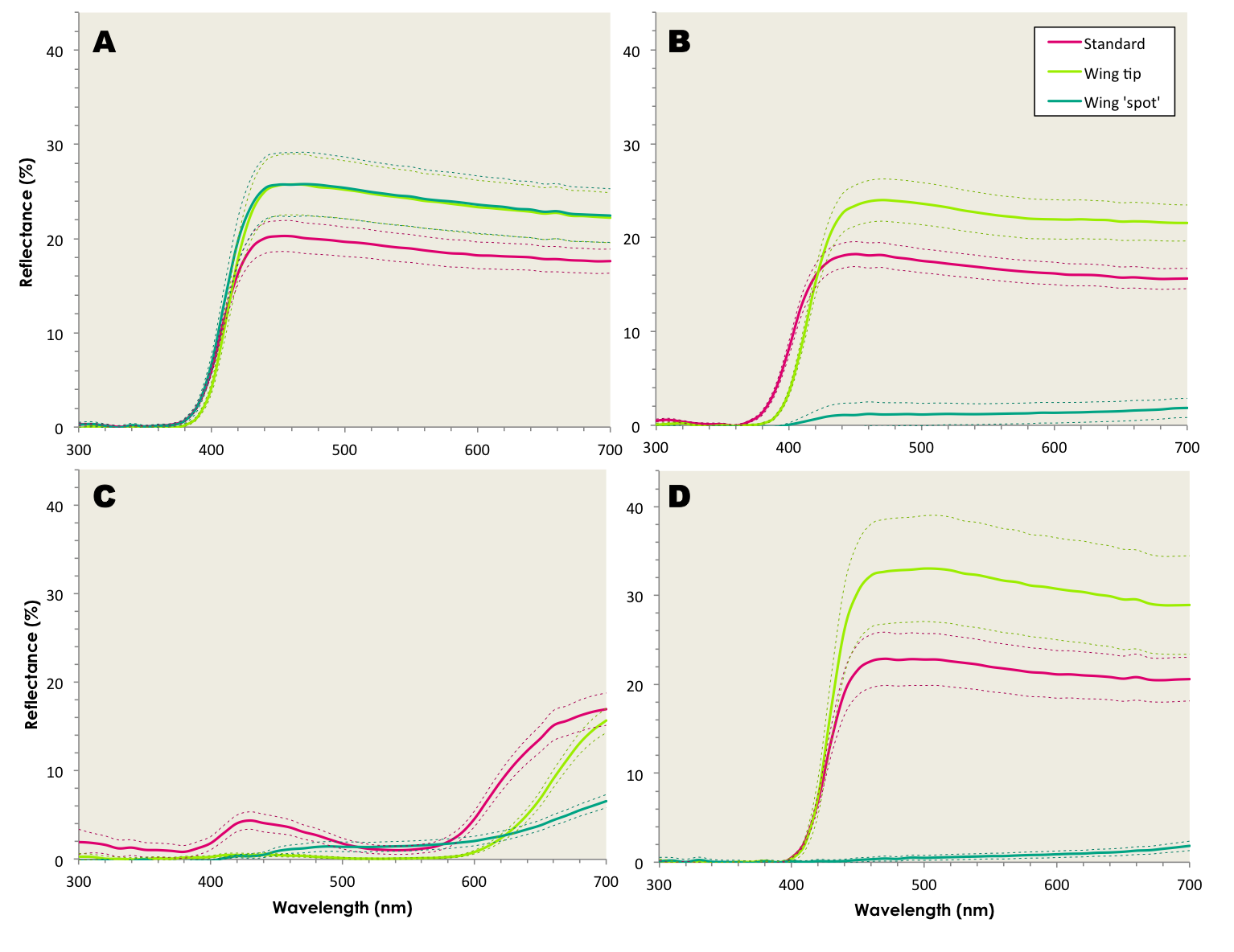


**Figure S8 –** The typical reflectance spectra of *V. faba* flowers. The reflectance of light by flowers in the bee visible range of 300nm to 700nm is given as a % compared to a pure white standard. Curves are coloured according to the position in the flower they were measured (standard petal = pink, wing tip = lime, and the center of the wing petal where the wing spot is usually located blue-green), solid lines give the average reflectance spectra of flowers, and the dotted lines the standard error (A) The spectra of NV643, a non-spotted flower (n = 15) (B) The spectra of NV641, a spotted flower (n = 20-21) (C) The spectra of crimson flowered NV706 (n = 11-12) (D) The spectra of the closed flower mutant NV658, this flower has a very similar bee-colour to typical spotted flowers, but is a cream colour to humans (n = 10-11).

**Table S9 -** The colour of the standard in *V. faba* flowers in bee-colour space. The average excitation (E) of UV, blue (B) and green (G) photoreceptors relative to a human green background are given, along with the x, y co-ordinates in bee-colour space that the excitation values correspond to. Measurements were made at the tip of the adaxial surface of the standard petal. The colour contrast of petals against a human-green background is also given.

| Line | n | Colour type | Photoreceptor excitation | | | Hexagon units | | Colour contrast |
| --- | --- | --- | --- | --- | --- | --- | --- | --- |
|  |  |  | E(UV) | E(B) | E(G) | x | y |  |
| NV020 | 11 | Spotted | 0.34 | 0.68 | 0.58 | 0.21 | 0.22 | 0.30 |
| NV027 | 10 | Spotted | 0.38 | 0.71 | 0.61 | 0.20 | 0.22 | 0.30 |
| NV079 | 12 | Spotted | 0.35 | 0.67 | 0.56 | 0.18 | 0.21 | 0.28 |
| NV082 | 11 | Spotted | 0.28 | 0.63 | 0.52 | 0.21 | 0.23 | 0.31 |
| NV100 | 11 | Spotted | 0.29 | 0.63 | 0.53 | 0.21 | 0.22 | 0.30 |
| NV129 | 10 | Spotted | 0.35 | 0.69 | 0.60 | 0.22 | 0.21 | 0.31 |
| NV155 | 11 | Spotted | 0.30 | 0.63 | 0.54 | 0.21 | 0.21 | 0.30 |
| NV175 | 10 | Non-spotted | 0.39 | 0.71 | 0.65 | 0.23 | 0.19 | 0.30 |
| NV293 | 19 | Spotted | 0.33 | 0.67 | 0.56 | 0.20 | 0.22 | 0.30 |
| NV490 | 10 | Spotted | 0.32 | 0.65 | 0.55 | 0.20 | 0.22 | 0.29 |
| NV574 | 9 | Spotted | 0.34 | 0.68 | 0.59 | 0.22 | 0.22 | 0.31 |
| NV604 | 11 | Spotted | 0.34 | 0.68 | 0.57 | 0.20 | 0.22 | 0.30 |
| NV619 | 15 | Spotted | 0.31 | 0.65 | 0.54 | 0.20 | 0.23 | 0.30 |
| NV620 | 11 | Spotted | 0.36 | 0.70 | 0.62 | 0.23 | 0.21 | 0.31 |
| NV626 | 11 | Spotted | 0.31 | 0.64 | 0.53 | 0.20 | 0.22 | 0.30 |
| NV639 | 15 | Spotted | 0.34 | 0.67 | 0.56 | 0.19 | 0.22 | 0.29 |
| NV640 | 11 | Spotted | 0.34 | 0.66 | 0.55 | 0.18 | 0.22 | 0.29 |
| NV641 | 20 | Spotted | 0.34 | 0.66 | 0.54 | 0.18 | 0.22 | 0.29 |
| NV643 | 15 | Spotted | 0.34 | 0.68 | 0.57 | 0.20 | 0.22 | 0.30 |
| NV644 | 13 | Non-spotted | 0.47 | 0.77 | 0.68 | 0.18 | 0.20 | 0.27 |
| NV648 | 11 | Spotted | 0.32 | 0.65 | 0.56 | 0.21 | 0.22 | 0.30 |
| NV649 | 10 | Spotted | 0.39 | 0.72 | 0.65 | 0.23 | 0.20 | 0.30 |
| NV650 | 11 | Spotted | 0.37 | 0.69 | 0.57 | 0.18 | 0.22 | 0.28 |
| NV653 | 12 | Spotted | 0.31 | 0.64 | 0.55 | 0.20 | 0.21 | 0.29 |
| NV658 | 11 | Spotted | 0.31 | 0.65 | 0.60 | 0.25 | 0.19 | 0.32 |
| NV671 | 12 | Spotted | 0.33 | 0.66 | 0.56 | 0.20 | 0.22 | 0.30 |
| NV673 | 11 | Spotted | 0.31 | 0.65 | 0.55 | 0.20 | 0.22 | 0.30 |
| NV675 | 14 | Spotted | 0.34 | 0.68 | 0.57 | 0.20 | 0.22 | 0.30 |
| NV676 | 11 | Non-spotted | 0.36 | 0.69 | 0.59 | 0.20 | 0.22 | 0.29 |
| NV706 | 11 | Crimson | 0.19 | 0.30 | 0.13 | -0.06 | 0.14 | 0.15 |

**Table S10** - The colour of the wing petal of *V. faba* flowers in bee-colour space. The average excitation (E) of UV, blue (B) and green (G) photoreceptors relative to a human green background are given, along with the x, y co-ordinates in bee-colour space that the excitation values correspond to. Measurements were made at the tip of the abaxial surface of the wing petal. The colour contrast of petals against a human-green background is also given.

| Line | n | Colour type | Photoreceptor excitation | | | Hexagon units | | Colour contrast |
| --- | --- | --- | --- | --- | --- | --- | --- | --- |
|  |  |  | E(UV) | E(B) | E(G) | x | y |  |
| NV020 | 11 | Spotted | 0.41 | 0.74 | 0.67 | 0.23 | 0.20 | 0.31 |
| NV027 | 10 | Spotted | 0.42 | 0.75 | 0.69 | 0.23 | 0.19 | 0.30 |
| NV079 | 12 | Spotted | 0.34 | 0.67 | 0.58 | 0.21 | 0.21 | 0.29 |
| NV082 | 10 | Spotted | 0.40 | 0.73 | 0.68 | 0.24 | 0.19 | 0.31 |
| NV100 | 11 | Spotted | 0.36 | 0.70 | 0.61 | 0.22 | 0.21 | 0.31 |
| NV129 | 10 | Spotted | 0.39 | 0.71 | 0.66 | 0.23 | 0.19 | 0.30 |
| NV155 | 11 | Spotted | 0.39 | 0.72 | 0.65 | 0.23 | 0.20 | 0.31 |
| NV175 | 10 | Non-spotted | 0.42 | 0.74 | 0.69 | 0.23 | 0.19 | 0.30 |
| NV293 | 19 | Spotted | 0.48 | 0.79 | 0.73 | 0.21 | 0.18 | 0.28 |
| NV490 | 10 | Spotted | 0.35 | 0.69 | 0.60 | 0.22 | 0.22 | 0.31 |
| NV574 | 9 | Spotted | 0.41 | 0.74 | 0.67 | 0.23 | 0.20 | 0.30 |
| NV604 | 11 | Spotted | 0.44 | 0.77 | 0.69 | 0.21 | 0.20 | 0.29 |
| NV619 | 14 | Spotted | 0.37 | 0.72 | 0.63 | 0.22 | 0.21 | 0.31 |
| NV620 | 11 | Spotted | 0.36 | 0.69 | 0.64 | 0.25 | 0.19 | 0.31 |
| NV626 | 11 | Spotted | 0.45 | 0.77 | 0.70 | 0.21 | 0.19 | 0.29 |
| NV639 | 15 | Spotted | 0.45 | 0.76 | 0.68 | 0.20 | 0.20 | 0.28 |
| NV640 | 14 | Spotted | 0.35 | 0.70 | 0.62 | 0.24 | 0.22 | 0.32 |
| NV641 | 20 | Spotted | 0.35 | 0.69 | 0.61 | 0.23 | 0.21 | 0.31 |
| NV643 | 15 | Spotted | 0.37 | 0.72 | 0.63 | 0.23 | 0.22 | 0.31 |
| NV644 | 14 | Non-spotted | 0.39 | 0.73 | 0.64 | 0.22 | 0.21 | 0.31 |
| NV648 | 10 | Spotted | 0.34 | 0.68 | 0.61 | 0.24 | 0.20 | 0.31 |
| NV649 | 10 | Spotted | 0.47 | 0.77 | 0.73 | 0.22 | 0.17 | 0.28 |
| NV650 | 12 | Spotted | 0.41 | 0.74 | 0.67 | 0.22 | 0.20 | 0.30 |
| NV653 | 12 | Spotted | 0.36 | 0.71 | 0.64 | 0.24 | 0.20 | 0.31 |
| NV658 | 10 | Spotted | 0.40 | 0.72 | 0.68 | 0.25 | 0.18 | 0.30 |
| NV671 | 12 | Spotted | 0.37 | 0.70 | 0.63 | 0.22 | 0.20 | 0.30 |
| NV673 | 11 | Spotted | 0.40 | 0.73 | 0.66 | 0.23 | 0.20 | 0.30 |
| NV675 | 14 | Spotted | 0.40 | 0.73 | 0.66 | 0.23 | 0.20 | 0.30 |
| NV676 | 11 | Non-spotted | 0.42 | 0.74 | 0.66 | 0.21 | 0.21 | 0.29 |
| NV706 | 13 | Crimson | 0.03 | 0.05 | 0.02 | -0.01 | 0.03 | 0.03 |

**Table S11** - The colour of the wing spot of *V. faba* flowers in bee-colour space. The average excitation (E) of UV, blue (B) and green (G) photoreceptors relative to a human green background are given, along with the x, y co-ordinates in bee-colour space that the excitation values correspond to. Measurements were made at the centre of the abaxial surface of the wing petal where the wing petal spot is usually located. The colour contrast of petals against a human-green background is also given.

| Line | n | Colour type | Photoreceptor excitation | | | Hexagon units | | Colour contrast |
| --- | --- | --- | --- | --- | --- | --- | --- | --- |
|  |  |  | E(UV) | E(B) | E(G) | x | y |  |
| NV020 | 11 | Spotted | -0.03 | -0.02 | 0.01 | 0.03 | -0.01 | 0.03 |
| NV027 | 10 | Spotted | -0.03 | -0.01 | 0.02 | 0.04 | -0.01 | 0.04 |
| NV079 | 10 | Spotted | -0.02 | 0.01 | 0.04 | 0.05 | 0.00 | 0.05 |
| NV082 | 10 | Spotted | -0.02 | -0.01 | 0.03 | 0.04 | -0.02 | 0.04 |
| NV100 | 11 | Spotted | -0.01 | 0.00 | 0.03 | 0.03 | -0.01 | 0.03 |
| NV129 | 10 | Spotted | 0.00 | 0.01 | 0.04 | 0.03 | -0.01 | 0.03 |
| NV155 | 10 | Spotted | -0.03 | 0.01 | 0.05 | 0.06 | 0.00 | 0.06 |
| NV175 | 10 | Non-spotted | 0.36 | 0.69 | 0.63 | 0.24 | 0.20 | 0.31 |
| NV293 | 19 | Spotted | -0.02 | 0.00 | 0.02 | 0.04 | 0.00 | 0.04 |
| NV490 | 10 | Spotted | -0.02 | 0.00 | 0.03 | 0.05 | 0.00 | 0.05 |
| NV574 | 9 | Spotted | 0.01 | 0.05 | 0.08 | 0.06 | 0.01 | 0.06 |
| NV604 | 10 | Spotted | -0.03 | -0.02 | 0.00 | 0.03 | -0.01 | 0.03 |
| NV619 | 15 | Spotted | -0.01 | 0.02 | 0.04 | 0.05 | 0.00 | 0.05 |
| NV620 | 11 | Spotted | 0.00 | -0.01 | 0.02 | 0.02 | -0.01 | 0.02 |
| NV626 | 11 | Spotted | -0.02 | -0.01 | 0.01 | 0.02 | -0.01 | 0.02 |
| NV639 | 15 | Spotted | 0.04 | 0.12 | 0.13 | 0.08 | 0.04 | 0.09 |
| NV640 | 12 | Spotted | -0.02 | -0.02 | 0.01 | 0.03 | -0.01 | 0.03 |
| NV641 | 21 | Spotted | 0.00 | 0.09 | 0.07 | 0.06 | 0.05 | 0.08 |
| NV643 | 15 | Spotted | 0.39 | 0.72 | 0.63 | 0.21 | 0.21 | 0.30 |
| NV644 | 14 | Non-spotted | 0.38 | 0.72 | 0.63 | 0.21 | 0.22 | 0.30 |
| NV648 | 10 | Spotted | -0.02 | -0.02 | 0.01 | 0.03 | -0.01 | 0.03 |
| NV649 | 10 | Spotted | -0.01 | -0.01 | 0.01 | 0.02 | -0.01 | 0.02 |
| NV650 | 11 | Spotted | -0.02 | 0.00 | 0.02 | 0.03 | 0.00 | 0.03 |
| NV653 | 12 | Spotted | 0.00 | 0.00 | 0.02 | 0.02 | -0.02 | 0.03 |
| NV658 | 11 | Spotted | 0.02 | 0.03 | 0.04 | 0.02 | 0.00 | 0.02 |
| NV671 | 10 | Spotted | -0.02 | -0.02 | 0.00 | 0.02 | -0.01 | 0.02 |
| NV673 | 10 | Spotted | -0.04 | -0.02 | -0.01 | 0.03 | 0.00 | 0.03 |
| NV675 | 14 | Spotted | -0.02 | 0.01 | 0.03 | 0.04 | 0.00 | 0.04 |
| NV676 | 11 | Non-spotted | 0.33 | 0.67 | 0.57 | 0.20 | 0.22 | 0.30 |
| NV706 | 12 | Crimson | 0.01 | 0.07 | 0.09 | 0.07 | 0.02 | 0.07 |

**Table S12 -** The predicted % spot size of *V. faba* wing petals by line based on the model standard_height = Line + Month + Replicate + Plant, where Plant was specified as a random effect. Group indicates the significance group lines are assigned in a Tukey-Kramer posthoc test, lines connected with the same letter within the same measurement are not significantly different at the α = 0.05 level. Table is ordered by % spot size.

| Line | Predicted value | SE | Lower 95% CL | Upper 95% CL | Group |
| --- | --- | --- | --- | --- | --- |
| NV706 | 20 | 1.0 | 18 | 22 | A |
| NV658 | 21 | 0.8 | 20 | 23 | A |
| NV574 | 24 | 0.9 | 22 | 26 | A |
| NV619 | 37 | 0.9 | 35 | 39 | B |
| NV079 | 38 | 1.0 | 36 | 40 | BC |
| NV639 | 39 | 0.8 | 37 | 40 | BCD |
| NV490 | 40 | 0.9 | 38 | 42 | BCDE |
| NV604 | 41 | 1.0 | 39 | 42 | BCDE |
| NV027 | 41 | 0.8 | 39 | 43 | BCDE |
| NV100 | 42 | 0.9 | 40 | 44 | CDEF |
| NV082 | 42 | 0.9 | 41 | 44 | CDEF |
| NV653 | 43 | 0.9 | 41 | 45 | DEF |
| NV155 | 43 | 0.9 | 41 | 45 | DEFG |
| NV129 | 43 | 0.9 | 42 | 45 | EF |
| NV293 | 44 | 1.0 | 42 | 46 | DEFGH |
| NV640 | 45 | 1.0 | 43 | 47 | EFGH |
| NV648 | 46 | 0.8 | 45 | 48 | FGHI |
| NV671 | 47 | 0.9 | 45 | 49 | FGHIJ |
| NV675 | 48 | 0.9 | 46 | 50 | GHIJ |
| NV626 | 48 | 0.9 | 47 | 50 | HIJ |
| NV673 | 48 | 0.9 | 47 | 50 | HIJ |
| NV620 | 50 | 1.0 | 49 | 52 | IJK |
| NV641 | 51 | 0.8 | 49 | 53 | JK |
| NV020 | 54 | 0.9 | 52 | 56 | K |
| NV649 | 54 | 0.7 | 53 | 56 | K |
| NV650 | 59 | 0.9 | 57 | 61 | L |

**Table S13 -** The predicted standard heights of *V. faba* flowers by line based on the model standard_height = Line + Month + Plant, where Plant was specified as a random effect. Group indicates the significance group lines are assigned in a Tukey-Kramer posthoc test, lines connected with the same letter within the same measurement are not significantly different at the α = 0.05 level. Table is ordered by standard height.

| Line | Predicted value | SE | Lower 95% CL | Upper 95% CL | Group |
| --- | --- | --- | --- | --- | --- |
| NV155 | 12 | 0.5 | 11 | 13 | A |
| NV620 | 12 | 0.5 | 11 | 13 | AB |
| NV129 | 14 | 0.4 | 13 | 14 | ABC |
| NV079 | 14 | 0.5 | 13 | 15 | ABCD |
| NV644 | 15 | 0.5 | 14 | 16 | ABCDEF |
| NV673 | 15 | 0.5 | 14 | 16 | BCDEFGH |
| NV490 | 15 | 0.5 | 14 | 16 | BCDEFG |
| NV640 | 15 | 0.5 | 14 | 16 | CDEFGH |
| NV604 | 15 | 0.5 | 14 | 16 | BCDEFGH |
| NV648 | 15 | 0.4 | 14 | 16 | CDE |
| NV641 | 15 | 0.4 | 15 | 16 | CDEFGHI |
| NV626 | 15 | 0.5 | 15 | 16 | CDEFGHI |
| NV675 | 16 | 0.5 | 15 | 17 | DEFGHIJ |
| NV082 | 16 | 0.4 | 15 | 17 | DEFGHIJ |
| NV100 | 17 | 0.5 | 16 | 18 | EFGHIJK |
| NV643 | 17 | 0.4 | 16 | 18 | EFGHIJK |
| NV671 | 17 | 0.5 | 16 | 18 | EFGHIJK |
| NV639 | 17 | 0.4 | 16 | 18 | GHIJK |
| NV020 | 17 | 0.5 | 16 | 18 | FGHIJK |
| NV574 | 17 | 0.5 | 17 | 18 | HIJK |
| NV706 | 18 | 0.5 | 17 | 19 | JK |
| NV676 | 18 | 0.6 | 17 | 19 | IJKL |
| NV619 | 19 | 0.5 | 18 | 20 | KL |
| NV653 | 19 | 0.5 | 18 | 20 | KL |
| NV293 | 19 | 0.5 | 18 | 20 | KLM |
| NV027 | 21 | 0.4 | 20 | 22 | LM |
| NV649 | 21 | 0.4 | 21 | 22 | M |
| NV650 | 22 | 0.5 | 21 | 23 | M |
| NV175 | 22 | 0.5 | 21 | 23 | M |

**Table S14 -** The predicted corolla tube length of *V. faba* flowers by line based on the model standard_height = Line + Month + Plant, where Plant was specified as a random effect. Group indicates the significance group lines are assigned in a Tukey-Kramer posthoc test, lines connected with the same letter within the same measurement are not significantly different at the α = 0.05 level. Table is ordered by corolla tube length

| Line | Predicted value | SE | Lower 95% CL | Upper 95% CL | Group |
| --- | --- | --- | --- | --- | --- |
| NV155 | 12 | 0.2 | 11 | 12 | A |
| NV100 | 12 | 0.2 | 12 | 13 | AB |
| NV673 | 13 | 0.2 | 12 | 13 | BC |
| NV640 | 13 | 0.2 | 13 | 14 | BCD |
| NV604 | 13 | 0.2 | 13 | 14 | BCD |
| NV643 | 13 | 0.2 | 13 | 14 | CDE |
| NV129 | 14 | 0.2 | 13 | 14 | CDEF |
| NV620 | 14 | 0.2 | 13 | 14 | CDEFG |
| NV675 | 14 | 0.2 | 13 | 14 | CDEFG |
| NV650 | 14 | 0.2 | 13 | 14 | CDEFG |
| NV639 | 14 | 0.2 | 13 | 14 | CDEFG |
| NV671 | 14 | 0.2 | 13 | 14 | CDEFGH |
| NV619 | 14 | 0.2 | 14 | 14 | DEFGH |
| NV641 | 14 | 0.2 | 14 | 14 | DEFGH |
| NV706 | 14 | 0.2 | 14 | 15 | DEFGH |
| NV574 | 14 | 0.2 | 14 | 15 | DEFGH |
| NV079 | 14 | 0.2 | 14 | 15 | DEFGH |
| NV082 | 14 | 0.2 | 14 | 15 | EFGHI |
| NV658 | 14 | 0.2 | 14 | 15 | FGHI |
| NV626 | 14 | 0.2 | 14 | 15 | EFGHI |
| NV293 | 14 | 0.2 | 14 | 15 | EFGHI |
| NV676 | 15 | 0.3 | 14 | 15 | EFGHI |
| NV490 | 15 | 0.2 | 14 | 15 | FGHI |
| NV644 | 15 | 0.2 | 14 | 15 | FGHI |
| NV653 | 15 | 0.2 | 14 | 15 | GHI |
| NV027 | 15 | 0.2 | 14 | 15 | GHI |
| NV020 | 15 | 0.2 | 14 | 15 | HI |
| NV649 | 15 | 0.2 | 15 | 16 | IJ |
| NV648 | 15 | 0.2 | 15 | 16 | IJ |
| NV175 | 16 | 0.2 | 16 | 17 | J |

**Table S15 -** The predicted wing area (mm^2^) of *V. faba* wing petals by line based on the model standard_height = Line + Month + Replicate + Plant, where Plant was specified as a random effect. Group indicates the significance group lines are assigned in a Tukey-Kramer posthoc test, lines connected with the same letter within the same measurement are not significantly different at the α = 0.05 level. Table is ordered by wing area.

| Line | Predicted value | SE | Lower 95% CL | Upper 95% CL | Group |
| --- | --- | --- | --- | --- | --- |
| NV155 | 60 | 3.2 | 53 | 66 | A |
| NV620 | 67 | 3.3 | 60 | 73 | AB |
| NV490 | 81 | 3.2 | 75 | 87 | BC |
| NV079 | 82 | 3.3 | 75 | 88 | BCD |
| NV100 | 88 | 3.0 | 82 | 94 | CDE |
| NV640 | 88 | 3.3 | 82 | 95 | CDE |
| NV673 | 91 | 3.2 | 85 | 97 | CDEF |
| NV675 | 95 | 3.1 | 89 | 101 | CDEFG |
| NV619 | 95 | 3.0 | 89 | 101 | CDEFG |
| NV082 | 98 | 2.9 | 93 | 104 | DEFGH |
| NV644 | 98 | 3.4 | 92 | 105 | DEFGHI |
| NV641 | 101 | 2.8 | 95 | 107 | EFGHI |
| NV653 | 103 | 3.1 | 97 | 109 | EFGHIJ |
| NV648 | 106 | 2.7 | 100 | 111 | FGHIJ |
| NV020 | 106 | 3.2 | 99 | 112 | FGHIJ |
| NV574 | 106 | 3.1 | 100 | 112 | FGHIJ |
| NV649 | 107 | 2.4 | 102 | 112 | GHIJ |
| NV293 | 107 | 3.3 | 100 | 114 | FGHIJ |
| NV604 | 108 | 3.3 | 101 | 114 | FGHIJ |
| NV643 | 108 | 2.8 | 102 | 113 | GHIJ |
| NV676 | 110 | 3.7 | 102 | 117 | GHIJ |
| NV639 | 111 | 2.8 | 105 | 116 | GHIJ |
| NV129 | 111 | 2.9 | 105 | 117 | GHIJ |
| NV027 | 112 | 2.9 | 107 | 118 | HIJ |
| NV626 | 115 | 3.1 | 109 | 121 | IJ |
| NV671 | 115 | 3.2 | 109 | 122 | IJ |
| NV658 | 118 | 2.9 | 112 | 124 | JK |
| NV706 | 133 | 3.2 | 127 | 140 | KL |
| NV175 | 150 | 3.3 | 144 | 157 | LM |
| NV650 | 160 | 3.0 | 154 | 166 | M |

**Table S16 –** Log (predicted wing length:width ratio) of *V. faba* wing petals by line based on the model standard_height = Line + Month + Plant, where Plant was specified as a random effect. Group indicates the significance group lines are assigned in a Tukey-Kramer posthoc test, lines connected with the same letter within the same measurement are not significantly different at the α = 0.05 level. Table is ordered by log(wing ratio).

| Line | Predicted value | SE | Lower 95% CL | Upper 95% CL | Group |
| --- | --- | --- | --- | --- | --- |
| NV155 | 0.66 | 0.02 | 0.62 | 0.71 | A |
| NV574 | 0.71 | 0.02 | 0.67 | 0.75 | AB |
| NV620 | 0.76 | 0.02 | 0.72 | 0.81 | ABC |
| NV020 | 0.77 | 0.02 | 0.73 | 0.81 | ABCD |
| NV640 | 0.78 | 0.02 | 0.74 | 0.83 | BCD |
| NV293 | 0.80 | 0.02 | 0.76 | 0.85 | BCDE |
| NV675 | 0.82 | 0.02 | 0.77 | 0.86 | BCDE |
| NV604 | 0.82 | 0.02 | 0.77 | 0.86 | BCDEF |
| NV641 | 0.82 | 0.02 | 0.79 | 0.86 | CDE |
| NV626 | 0.84 | 0.02 | 0.80 | 0.88 | CDEFG |
| NV619 | 0.85 | 0.02 | 0.81 | 0.89 | CDEFG |
| NV079 | 0.85 | 0.02 | 0.81 | 0.89 | CDEFGH |
| NV129 | 0.86 | 0.02 | 0.82 | 0.89 | CDEFGH |
| NV082 | 0.86 | 0.02 | 0.82 | 0.90 | CDEFGH |
| NV643 | 0.86 | 0.02 | 0.82 | 0.90 | CDEFGH |
| NV649 | 0.87 | 0.02 | 0.83 | 0.90 | DEFGH |
| NV100 | 0.87 | 0.02 | 0.83 | 0.91 | CDEFGH |
| NV676 | 0.88 | 0.02 | 0.83 | 0.93 | CDEFGHI |
| NV658 | 0.90 | 0.02 | 0.87 | 0.94 | EFGHI |
| NV650 | 0.90 | 0.02 | 0.87 | 0.94 | EFGHI |
| NV644 | 0.92 | 0.02 | 0.87 | 0.96 | EFGHIJ |
| NV639 | 0.93 | 0.02 | 0.89 | 0.97 | FGHIJ |
| NV027 | 0.93 | 0.02 | 0.90 | 0.97 | GHIJ |
| NV653 | 0.96 | 0.02 | 0.92 | 1.00 | HIJ |
| NV673 | 0.96 | 0.02 | 0.91 | 1.00 | HIJ |
| NV175 | 0.98 | 0.02 | 0.94 | 1.02 | IJK |
| NV648 | 0.99 | 0.02 | 0.95 | 1.02 | IJK |
| NV490 | 1.02 | 0.02 | 0.98 | 1.07 | JKL |
| NV671 | 1.08 | 0.02 | 1.04 | 1.13 | KL |
| NV706 | 1.12 | 0.02 | 1.08 | 1.16 | L |

**Table S17** - The absolute amount of VOCs produced by abscised flowers of lines NV641 and NV676 of *V. faba*. The predicted compounds for each peak based on their mass spectra are given. Peaks also identified in a negative control of air only were excluded unless the peak in the sample of interest was appreciably higher. The compounds at the retention time of 12.44, 12.79 and 13.8 were predicted to be one of two compounds with equal likelihood and therefore both are given. *The presence of ocimene (retention time = 8.09) in the VOCs of line NV641 was confirmed by co-collection with flowers of line NV676. The compound at a retention time of 7.95 was predicted to be pinene or ocimene, however, the retention times of authentic standards did not match this compound, and therefore the compound with a retention time of 7.95 is an as of yet unidentified monoterpene.

| **Retention time (RT)** | **Predicted compound** | **Amount of volatiles produced (µg ocimene equivalents)** | |
| --- | --- | --- | --- |
|  |  | **NV641** | **NV676** |
| 7.74 | **Limonene** | 1.1 (± 1.1) | 0.0 (± 0.0) |
| 7.95 | **Unidentified monoterpene*** | 1.1 (± 0.7) | 0.0 (± 0.0) |
| 8.09 | **Ocimene*** | 51.3 (± 19.9) | 0.0 (± 0.0) |
| 8.76 | **Linalool** | 12.5 (± 7.5) | 20.5 (± 9.0) |
| 9.26 | **Myrtenol** | 0.2 (± 0.1) | 0.0 (± 0.0) |
| 10.57 | **Cinnamaldehyde** | 0.2 (± 0.1) | 0.2 (± 0.1) |
| 10.92 | **Cinnamyl alcohol** | 1.3 (± 1.3) | 1.9 (± 0.7) |
| 11.92 | **Caryophyllene** | 5.7 (± 3.5) | 11.4 (± 3.6) |
| 12.14 | **Cinnamyl acetate** | 0.1 (± 0.1) | 0.0 (± 0.0) |
| 12.21 | **Humulene** | 1.4 (± 0.8) | 1.7 (± 0.6) |
| 12.45 | **Copaene/Germacrene** | 3.1 (± 1.8) | 2.1 (± 1.1) |
| 12.66 | **Farnesene** | 1.0 (± 0.7) | 0.0 (± 0.0) |
| 12.79 | **Copanene/Napthalene** | 0.8 (± 0.3) | 0.7 (± 0.2) |
| 13.26/46 | **Caryophyllene oxide** | 0.5 (± 0.3) | 0.6 (± 0.2) |
| 13.8 | **Cadinol/murrolol** | 0.0 (± 0.0) | 0.2 (± 0.1) |
